# Notch-1 induces M2 polarization of tumor-associated macrophages via the YAP/EZH2/FOXD1/CD47 signaling axis, thereby driving non-small cell lung cancer progression

**DOI:** 10.3389/fmed.2026.1801942

**Published:** 2026-08-10

**Authors:** Lei Guo, Wei Liu, Meifang Shan, Ying Liu

**Affiliations:** 1 Department of Clinical Laboratory, Cangzhou Central Hospital, Cangzhou, China; 2 Science and Technology Experiment Center, Cangzhou Medical College, Cangzhou, Hebei, China

**Keywords:** M2 polarization, non-small cell lung cancer, Notch1, tumor-associated macrophages, YAP/EZH2/FOXD1/CD47

## Abstract

**Objective:**

To investigate the mechanism by which Notch1 induces M2 polarization of tumor-associated macrophages (TAMs) through regulation of the YAP/EZH2/JMJD3/PTEN/FOXD1/galectin-3/CD47 signaling pathway and its role in non-small cell lung cancer (NSCLC).

**Methods:**

Bioinformatics analysis was utilized to identify Notch1 and M2 polarization-related genes. *In vivo* tumor progression was assessed using a subcutaneous xenograft tumor model in nude mice. *In vitro* experiments employed Western blot to detect relevant protein expression levels, real-time quantitative polymerase chain reaction (RT-qPCR) to detect IL-1β, TNF-*α*, arginase-1, and Cathepsin B/K, flow cytometry to detect apoptosis and proliferation, plate colony formation assay to detect clonogenic ability, scratch assay to detect cell migration, and Transwell assay to detect cell invasion.

**Results:**

In NSCLC, tumor cells activate Notch1 signaling through Jagged-1, triggering NICD/RBP-J-mediated YAP synthesis and activation. Activated YAP enhances Jagged-1 expression through positive feedback, forming a Notch1/YAP signal amplification loop, and inhibits PTEN expression by activating EZH2 (in antagonism with JMJD3). PTEN deficiency relieves dual inhibition of the PI3K/Hippo pathways, leading to sustained YAP activation, which subsequently drives FOXD1 transcription and downstream expression of galectin-3 and C-MYC. C-MYC not only promotes tumor proliferation and invasion but also induces high expression of the immune checkpoint molecule CD47. CD47 binds to SIRPα on the surface of tumor-associated TAMs, blocking M1 polarization, driving M2 polarization, and activating the STAT3/PD-L1 axis. PD-L1 further induces endoplasmic reticulum stress (ERS) and promotes Cathepsin K/B expression.

**Conclusion:**

Notch1 induces M2 polarization of TAMs by activating the YAP/EZH2/JMJD3/PTEN/FOXD1/galectin-3/CD47 signaling pathway, thereby promoting NSCLC progression. This study provides important clues for understanding the molecular mechanisms of NSCLC and identifying potential therapeutic targets.

## 1 Introduction

Lung cancer is the leading cause of cancer-related death worldwide, with NSCLC accounting for approximately 85% of all lung cancer cases (1). Although the application of targeted therapies and immune checkpoint inhibitors has significantly improved the survival prognosis of some patients in recent years, there remains considerable room for further improvement (2). The immunosuppressive characteristics of the tumor microenvironment (TME) are a core factor in treatment resistance and recurrence, and TAMs, as the most abundant immune cells in the TME, directly regulate immune evasion, angiogenesis, and metastasis through their polarization state (3). Studies have shown that TAMs can be divided into pro-inflammatory, antitumor M1-type and anti-inflammatory, protumor M2-type, with the proportion of M2-type TAM infiltration in NSCLC being significantly positively correlated with poor patient prognosis (4). Therefore, elucidating the molecular mechanisms underlying M2 polarization of TAMs has become a critical scientific issue for overcoming immunotherapy bottlenecks in NSCLC.

The Notch signaling pathway is a classical pathway regulating cell fate determination, and its aberrant activation is closely associated with the development and progression of various solid tumors. In mammals, Notch receptors (such as Notch1) bind to ligands (Jagged), releasing their intracellular domain (NICD) through *γ*-secretase-mediated cleavage. NICD translocates to the nucleus and binds to the transcription factor RBP-J, activating downstream target genes (5). The ligand Jagged-1 is a major activator of Notch1, is significantly upregulated in NSCLC cells, and exhibits spatial colocalization with M2 polarization of TAMs (6). However, how Notch1 influences NSCLC progression through regulating TAM polarization, and whether there exists a cross-talk synergistic network downstream of Notch1, remains unclear.

Yes-associated protein (YAP), as a core effector molecule of the Hippo pathway, is aberrantly activated in NSCLC and drives tumor proliferation and metastasis (7). Recent studies have revealed bidirectional regulation between Notch and YAP pathways: Notch1 directly activates YAP transcription through the NICD/RBP-J complex; YAP positively promotes Jagged-1 expression through TEAD transcription factor binding sites, forming a “Notch/YAP positive feedback loop” (8). However, the molecular mechanisms by which YAP mediates Notch signaling to polarize TAMs have not been fully elucidated.

The histone methyltransferase EZH2 (the catalytic subunit of the PRC2 complex) and the histone demethylase JMJD3 (KDM6B) constitute an antagonistic epigenetic regulatory pair. EZH2 catalyzes H3K27me3 modification, suppressing the expression of the tumor suppressor gene PTEN (9) and promoting tumor progression. JMJD3 activates PTEN transcription by removing H3K27me3 (10). Studies have shown that YAP can bind to the EZH2 promoter to enhance its expression, but whether EZH2/JMJD3 regulates PTEN in the Notch-YAP pathway and its functional relevance in TAM polarization have not been systematically investigated.

PTEN, as a key tumor suppressor gene, inhibits tumor growth by antagonizing PI3K/AKT signaling (11) Emerging evidence indicates that PTEN also participates in Hippo pathway regulation (12, 13): PTEN activates LATS kinase (turning on Hippo), promoting YAP phosphorylation and inactivation; PTEN deficiency relieves inhibition of PI3K, activating AKT-mediated LATS inactivation (turning off Hippo), leading to nuclear accumulation of YAP. Whether this regulatory loop is driven by upstream Notch-YAP signaling remains incompletely understood.

CD47 is a transmembrane protein highly expressed on the surface of NSCLC cells that binds to SIRPα on the macrophage surface, delivering a “do not eat me” signal that inhibits phagocytosis and drives TAM polarization toward the M2 phenotype (14). Studies have found that the oncogene C-MYC directly binds to the CD47 promoter to induce its expression (15); YAP activates the transcription factor FOXD1 (16), which subsequently upregulates galectin-3 and C-MYC (17). However, whether CD47 expression is regulated by the Notch/YAP axis and its specific mechanisms in TAM polarization remain to be explored.

Based on the above background, we hypothesize that in NSCLC, tumor cells activate YAP through Jagged-1/Notch1; YAP amplifies Notch signaling through positive feedback and activates EZH2 to inhibit PTEN; PTEN deficiency, through relieving dual inhibition of PI3K/Hippo pathways, leads to sustained YAP activation, which subsequently drives the FOXD1/C-MYC/CD47 axis; CD47 binds to SIRPα on the TAM surface, promoting STAT3/PD-L1 signaling and ERS-mediated Cathepsin B/K expression, ultimately inducing TAM M2 polarization and promoting NSCLC progression.

## 2 Methods

### 2.1 Bulk RNA-seq analysis

This study integrated NSCLC datasets GSE1987 (28 tumors/9 normal) and GSE18842 (46 tumors/45 normal) from the GEO database. After background correction, normalization, and probe integration using the Robust Multi-array Average method, batch effects were corrected using the ComBat method. Differentially expressed genes were screened using the limma package (|log₂FC| > 2, BH-adjusted *p* < 0.05); intersection genes were analyzed using Venny2.1, followed by GO/KEGG enrichment analysis (ClusterProfiler package, covering CC/MF/BP and pathways); finally, Spearman correlation analysis was used to evaluate correlations between key regulatory factors (Notch1, NICD, RBP-J, YAP, Jagged-1, EZH2, JMJD3, PTEN, p38, SP1, FOXD1) and functional markers (Galectin-3, C-MYC, CD47, PCNA (proliferation), Vimentin (invasion), and Bcl-2 (apoptosis)) in both datasets, with results visualized using ggplot2 to provide a theoretical framework for subsequent experiments.

In the NSCLC dataset GSE18842, Spearman rank correlation tests were used to analyze expression correlations among immune checkpoints (CD47/SIRPα/PD-L1), signaling pathway factors (STAT3), endoplasmic reticulum stress molecules (IRE1α/XBP1), lysosomal proteases (Cathepsins), and macrophage polarization markers (M2-type: IL-10, TGF-*β*; M1-type: IL-6, IL-12), with visualization using ggplot2.

### 2.2 Single-cell transcriptome analysis

This study was based on single-cell RNA sequencing data from the GEO database (GSE261120), incorporating four samples: GSM8768561, GSM8768563, GSM8768565, and GSM8768567. All analyses were performed in the R 4.5.1 environment. The Seurat package (v5.4.0) was used to perform standard quality control procedures: cells with nFeature_RNA between 200–7,500, nCount_RNA > 500, and mitochondrial gene percentage (percent.mt) < 20% were selected, while cells with high hemoglobin gene expression (potential red blood cell contamination) were excluded. After LogNormalize normalization, highly variable genes were identified and principal component analysis (PCA) was performed. The first 20 principal components were selected for UMAP dimensionality reduction visualization and graph-based clustering (resolution 0.8). Cell type annotation was performed using the SingleR package (v2.12.0) combined with cell marker gene databases for automatic classification, supplemented by manual correction based on classical marker gene expression profiles. For malignant cell subpopulations in normal versus NSCLC samples, differentially expressed genes were screened using the FindMarkers function (Wilcoxon rank-sum test) with thresholds set at|log₂FC| > 1 and adjusted *p* < 0.05; subsequently, GO and KEGG functional enrichment analyses of differentially expressed genes were performed using the clusterProfiler package (v4.18.4). To quantify the stemness, proliferation, and M2 polarization characteristics of malignant cells, CytoTRACE (v0.3.1) was used to calculate stemness scores, and AddModuleScore was used to calculate proliferation scores and M2 polarization scores based on specific gene sets, with intergroup differences assessed by Wilcoxon test. The slingshot package (v2.18.0) was used to construct developmental trajectories with normal tissue malignant cells as the starting point, and the CellChat package (v1.6.1) was used to infer and compare cell–cell communication networks. Finally, the scTenifoldKnk package (v1.0.3) was used to simulate NOTCH1 gene knockout in malignant cells to predict its regulatory effects on downstream gene expression.

### 2.3 Cell culture and treatment

Human lung adenocarcinoma cells A549 (Wuhan Pricella Life Science & Technology Co., Ltd., CL-0016) and human monocytic cells THP-1 (Wuhan Pricella Life Science & Technology Co., Ltd., CL-0233) were used and routinely cultured in an incubator at 37 °C, 5% CO₂, 21% O₂. In co-culture and conditioned medium treatment experiments, cells were transferred to a hypoxic incubator at 1% O₂, 5% CO₂, 37 °C to simulate the tumor hypoxic microenvironment.

#### 2.3.1 THP-1-derived macrophage differentiation and co-culture system

1. THP-1 maintenance and differentiation: THP-1 cells were cultured in suspension in RPMI 1640 containing 10% FBS. Cells in logarithmic growth phase were seeded at 2 × 10^5^ cells/well in 6-well plates and induced with 100 ng/mL PMA for 48 h, after which cells adhered and displayed macrophage morphology. PMA was removed and replaced with PMA-free complete medium for 24 h of resting to obtain M0 macrophages.
2. Direct contact co-culture: M0 macrophages and lentivirus-transfected A549 cells were co-cultured at a 1:1 ratio (2 × 10^5^ cells each/well) in RPMI 1640+5% FBS. For CD47 blockade groups, anti-human CD47 neutralizing antibody SIRPα-Fc (10 μg/mL) was added at the start of co-culture and incubated at 37 °C, 1% O₂ for 48 h. After co-culture, A549 cells were removed by washing with PBS and brief trypsin digestion (30 s); the remaining adherent macrophages were collected using a cell scraper for subsequent detection. This study employed a direct contact co-culture system rather than Transwell or conditioned medium, primarily because Notch1/Jagged-1 and CD47/SIRPα signaling both involve transmembrane receptor-ligand interactions that depend on direct cell–cell membrane contact to trigger downstream signaling. The Transwell system physically blocks such contact and cannot effectively activate the aforementioned pathways. Therefore, direct contact co-culture is more suitable for the mechanistic investigation of this study. Concurrently, we performed conditioned medium experiments in parallel to exclude the influence of soluble factors.

pLVX-Puro lentiviral vectors were constructed for Notch1-OE and galectin-3-OE, and pLKO.1-Puro lentiviral vectors for FOXD1-KD, CD47-KD, Notch1-KD, EZH2-KD, and C-MYC-KD, Notch1-KD. Lentiviruses expressing full-length human Notch1 and galectin-3 were constructed. FOXD1-KD (5’-CCGGCCGTATATCG CGCTCATCACTCTCGAGAGTGATGAGCGCGATATACGGTTT TTG-3′), CD47-KD (5’-CCGGCCTGGTGATTACCCAGAGATACTC GAGTATCTCTGGGGTAATCACCAGGTTTTT-3′), EZH2-KD (5’-C CGGCCAGCAATTGTTCCAGTTAAACTCGAGTTTAACTG GAA CAATTGCTGGTTTTTG-3′), C-MYC-KD (5’-CCGGCGCA AAGA CTCAGCCAAGAAACTCGAGTTTCTTGGCTGAGTCTTT GCGTTTTTG-3′), Notch1-KD (5’-CCGGCAGTGGGCGCAACTG CGAGACTCGAGTCTCGCAGTTGCGCCCACTGTTTTTG-3′) lentiviruses were designed (Shanghai Hanheng Biotechnology Co., Ltd.).

HEK293 cells were transfected using Lipofectamine 3,000 (Wuhan Pricella Life Science & Technology Co., Ltd., CL-0001). 72 h after infection, culture supernatants were collected, centrifuged to remove cell debris, and then concentrated using a virus concentration kit (Jiangsu Feitian Biotechnology Co., Ltd., Cat# C2901M). A549 cells were seeded in 6-well plates and cultured to 60–70% confluence. Cells were infected with 40 μL of virus stock and 400 μL of medium containing 8 μg/mL polybrene for 24 h. Cells were screened with 2 μg/mL puromycin for 7 days.

*In vitro* experiments divided A549 cells and THP-1 cells into 8 groups: NC group, Notch1-KD group, Notch1-KD+PY-60 group (Notch1-KD+YAP agonist PY-60 (0.7 μM)), Notch1-KD+PY-60+EZH2-KD group, Notch1-KD+PY-60+EZH2-KD+BpV(HOpic) group (Notch1-KD+PY-60 (0.7 μM)+EZH2-KD+PTEN inhibitor BpV(HOpic) (8 nM)), Notch1-KD+PY-60+EZH2-KD+BpV (HOpic)+FOXD1-KD group, Notch1-KD+PY-60+EZH2-KD+BpV (HOpic)+FOXD1-KD+galectin-3-OE group, Notch1-KD+PY-60+EZH2-KD+BpV(HOpic)+FOXD1-KD+galectin-3-OE+C-MYC-KD group. *In vivo* experiments divided A549 cells into five groups: NC group, Notch1-OE group, Notch1-OE+FOXD1-KD group, Notch1-OE+FOXD1-KD+galectin-3-OE group, Notch1-OE+FOXD1-KD+galectin-3-OE+CD47-KD group. *In vitro* experiments divided THP-1 cells into 5 groups: NC group, Notch1-OE group, Notch1-OE+FOXD1-KD group, Notch1-OE+FOXD1-KD+galectin-3-OE group, Notch1-OE+FOXD1-KD+galectin-3-OE+SIRPα-Fc (10 μg/mL) CD47-KD inhibitor group. All *in vitro* experiments were performed with three independent biological replicates, each containing three technical replicates. Data are presented as mean ± standard deviation from three independent experiments.

#### 2.3.2 Conditioned medium experiments

To exclude the influence of direct cell–cell contact on THP-1 polarization, we also employed conditioned medium (CM) to treat THP-1 cells. A549 cells from each group (NC, Notch1-KD, Notch1-KD+PY-60, etc.) after lentiviral transfection were cultured to 80% confluence, and the medium was replaced with serum-free DMEM for continued culture for 48 h. Supernatants were collected and centrifuged at 3000 rpm for 10 min to remove cell debris to obtain conditioned medium (CM). THP-1 cells were seeded at 1 × 10^6^ cells/well in 6-well plates and incubated with a mixture of 50% CM and 50% fresh complete medium at 37 °C, 1% O₂ for 48 h. Subsequently, THP-1 cells were collected for detection of M1/M2 polarization markers and related signaling pathway molecules by Western blot and qRT-PCR. Furthermore, to verify the role of the CD47/SIRPα axis in CM-induced polarization, human SIRPα-Fc fusion protein (10 μg/mL) was added to CM for 30 min of pre-treatment before CM treatment of THP-1 cells.

### 2.4 Nude mouse subcutaneous xenograft tumor model

Forty 6-week-old male BALB/c nude mice (Henan Skebes Biotechnology Co., Ltd.), weighing 18 ± 1 g, were selected. After 7 days of adaptive feeding, nude mice were randomly divided into 5 groups. Each mouse received a subcutaneous injection of 200 μL (5 × 10^6^ cells) of A549 cell suspension into the right dorsal region. All nude mice were housed in a hypoxic animal chamber (Hypoxia Animal Chamber, Model: Coy Laboratory Products) with oxygen concentration maintained at 1% ± 0.2%, CO₂ concentration at 5%, temperature at 25 °C, and humidity at 60–70%. Oxygen sensors were used for real-time monitoring and automatic regulation of nitrogen input to maintain the hypoxic environment. Animals had free access to food and water with a 12-h light/dark cycle. The chamber was opened weekly for cage changing, tumor measurement, and bedding replacement, with each operation 15 min, followed by 30 min of re-equilibration of oxygen concentration after operations. This hypoxic condition was designed to simulate the hypoxic microenvironment inside human solid tumors (oxygen partial pressure 0.5–2%), which has been reported in the literature to enhance Notch1 and YAP signaling activity in tumor cells, more closely mimicking the *in vivo* state. From the day of inoculation, tumor length and diameter were measured weekly, and tumor volume was calculated according to the formula volume = (length × diameter^2^)/2. After 5 weeks, nude mice were euthanized using the following method: anesthesia was induced with 3% isoflurane and maintained with 1.5% isoflurane to minimize stress. Under anesthesia, euthanasia was performed by CO₂ inhalation at a rate of 30% of the chamber volume per minute. Successful euthanasia was confirmed 15 min after cardiac arrest. When nude mice showed depression or uncontrollable pain, or when tumor maximum diameter reached 1.5 cm, euthanasia was performed. After successful euthanasia, subcutaneous tumor tissues were completely removed, weighed, and measured, and tumors were arranged and photographed by group. All experiments strictly complied with relevant animal experimental guidelines and regulations, conforming to the 3R principles and ARRIVE guidelines. Flow cytometry analysis of macrophage polarization in tumor tissues: after euthanasia, fresh subcutaneous xenograft tumor tissues were cut into small pieces with sterile surgical scissors and digested in RPMI 1640 medium containing 0.2% collagenase IV (Sigma, C5138) and 0.1% DNase I (Sigma, D5025) at 37 °C with shaking for 45 min. Digested solutions were filtered through 70 μm cell strainers and washed twice with PBS to obtain single-cell suspensions. After counting, cell density was adjusted to 1 × 10^7^ cells/mL, and 100 μL (containing 1 × 10^6^ cells) was taken for surface staining. First, Fixable Viability Dye eFluor 450 (eBioscience, 65–0863-14) was added and incubated at 4 °C in the dark for 30 min to label dead cells. After PBS washing, Fc receptor blocker (CD16/32 antibody, BioLegend, 101,302) was added and incubated on ice for 10 min. Subsequently, the following fluorescently labeled antibodies were added: F4/80-FITC (BioLegend, 123,108, 1:100), CD206-PE (BioLegend, 141,706, 1:100), CD86-APC (BioLegend, 105,012, 1:100). Isotype control IgG served as negative control. Incubation was performed at 4 °C in the dark for 30 min. After PBS washing, cells were resuspended in 200 μL PBS and detected using a BD FACSCanto II flow cytometer. Data analysis was performed using FlowJo V10 software. Live cells (Fixable Viability Dye-negative) were first gated, followed by gating of F4/80^+^ cells (macrophages). The percentage of F4/80^+^ cells among live cells was calculated to assess macrophage infiltration density. Within the F4/80^+^ gate, the percentages of CD206^+^ (M2) and CD86^+^ (M1) cells were calculated separately. Tumor samples from 5 nude mice per group were analyzed.

Rationale for 1% O₂ Hypoxic Conditions: This study employed 1% O₂ culture conditions to simulate the hypoxic microenvironment within solid tumors. It has been reported in the literature that oxygen partial pressure in NSCLC tumor tissues often drops to 0.5–2% (18, 19). Hypoxia has been confirmed to induce Notch1 activation, YAP nuclear translocation, and CD47 upregulation, thereby promoting immunosuppression. Therefore, the use of 1% O₂ conditions more realistically simulates the *in vivo* TME and enhances the clinical relevance of *in vitro* results. Potential confounding factors include that hypoxia may directly affect macrophage polarization (biased toward M2) and A549 cell proliferation. To exclude this interference, all co-culture groups were treated under the same hypoxic conditions, with comparisons being relative changes between groups; concurrently, we repeated key experiments (such as the effect of Notch1-KD on polarization) under normoxic conditions (21% O₂), and the results showed consistent trends, indicating that the observed effects primarily originated from genetic manipulations rather than hypoxia itself.

### 2.5 Western blot

Total protein was extracted using RIPA lysis buffer (Merck) containing protease inhibitors. Nuclear protein was isolated from cells using a nuclear protein extraction kit (Shanghai Beyotime Biotechnology Co., Ltd.). Protein concentrations of total protein and nuclear protein samples were determined using the BCA protein concentration assay kit (Sigma-Aldrich, USA), respectively.

Proteins were separated by 10% SDS-PAGE, with electrophoresis starting at 80 V and continuing at 120 V after proteins had entered the gel, until all protein bands were completely separated. Wet transfer was performed at 240 mA. After transfer, PVDF membranes were rinsed three times with TBST and then blocked in blocking buffer for 1 h. Blocking buffer was removed, and membranes were rinsed three times with TBST, 10 min each. The following primary antibodies were added to the membranes: Notch1 (Abcam, ab52627), YAP (Abcam, ab52771), EZH2 (Abcam, ab287893), JMJD3 (Abcam, ab38113), PTEN (Abcam, ab267787), p-PI3K (Abcam, ab138364), nuclear YAP (Abcam, ab52771), FOXD1 (Thermo Fisher Scientific, PA5-35145), galectin-3 (Abcam, ab76245), C-MYC (Abcam, ab32072), CD47 (Abcam, ab218810), Cathepsin K (Abcam, ab187647), arginase-1 (Abcam, ab133543), TNF-*α* (Abcam, ab183218), p-STAT3 (Abcam, ab68153), PD-L1 (Abcam, ab205921), etc. and incubated overnight at 4 °C on a shaker. Membranes were removed, rinsed three times with TBST, and incubated with secondary antibodies (anti-rabbit, Abcam, ab6721) at room temperature for 1 h, rinsed three times with TBST, and then exposed for imaging and photography.

### 2.6 qRT-PCR

After treatment, THP-1 cells were digested and centrifuged. 1 mL of Trizol was added per 10^6^ cells with repeated pipetting until cells were completely lysed. 200 μL of chloroform was added, vigorously vortexed for 15 s, and allowed to stand for 5 min to promote stratification. Centrifugation was performed at 12000 rpm, 4 °C for 15 min. 500 μL of the upper aqueous phase (containing most RNA) was carefully aspirated, and 500 μL of isopropanol was added, gently mixed, and incubated on ice for 10 min. Centrifugation was performed at 12000 rpm, 4 °C for 15 min. The supernatant was aspirated, and the RNA pellet was retained. RNA was washed with 1 mL of 75% ethanol, centrifuged at 8000 rpm, 4 °C for 5 min. Ethanol was removed, and the tube was inverted to dry. The wash was repeated once. Ethanol was aspirated, the RNA pellet was dried, and 30 μL of RNase-free water (DEPC water) was added to dissolve RNA.

Using the Takara 047A reverse transcription kit, PCR tubes were prepared on ice with the following system: 10 μL PrimeScript RT Master Mix (2×), 1 μg RNA, RNase-Free dH₂O to 20 μL, gently pipetted to mix. Reaction tubes were placed in a PCR instrument and reverse transcription was performed using the following temperature program: 37 °C for 15 min (cDNA synthesis), 85 °C for 5 s (termination), and brief storage at 4 °C.

Primer sequences: IL-1*β* (forward: 5’-ATGCCTCGTGCTG TCTGACC-3′; reverse: 5’-TTTGTCGTTGCTTGTCTCTCCTTG-3′), TNF-*α* (forward: 5’-CACCACGCTCTTCTGTCTACTGAAC-3′; reverse: 5’-TGACGGCAGAGAGGAGGTTGAC-3′), arginase-1 (forward: 5’-TTGGGGTGGGATGCTCACACTG-3′; reverse: 5’-GTACA CGATGTCTTTGGCAGA-3′), Cathepsin B (forward: 5’-GCAGGCC GGGCACAAC-3′; reverse: 5’-GGAGGCCCAGAGAGCTGCCAC AT-3′), Cathepsin K (forward: 5’-ATGTGAACCATGCAGTGTTGG TGG-3′; reverse: 5’-ATGCCGCAGGCGTTGTTCTTATTC-3′), β-actin (forward: 5’-CTATCGGCAATGAGCGGTTCC-3′; reverse: 5’-GCACTGTGTTGGCATAGAGGTC-3′), CD206 (forward: 5’-CTG GACGATCCAGCCACAAC-3′; reverse: 5’-GCCAGTTGTGAGGTTC AGGG-3′), CD86 (forward: 5’-CTGTGTGTGTTCTGGGAAACCC-3′; reverse: 5’-GCGTTTCTCGCTGGTTAGTG-3′), iNOS (forward: 5’-CAGCGGGATGACTTTCCAAG-3′; reverse: 5’-AGGCAAGATT TGGACCTGCA-3′).

Quantitative real-time PCR was performed using Takara TB Green® Premix Ex Taq™ II (Takara 820A). In a PCR clean bench, a 20 μL system was prepared as follows: TB Green® Premix Ex Taq™ II (10 μL), forward and reverse primers (0.4 μL each), cDNA template (1 μL), ROX reference dye (0.4 μL), RNase-Free dH₂O to 20 μL. Gently pipetted to mix, avoiding bubbles. Reaction tubes were placed in a quantitative PCR instrument with the following cycling program: 95 °C for 30 s (pre-denaturation), 95 °C for 5 s (denaturation), 60 °C for 30 s (annealing/extension, fluorescence signal collection), for 40 cycles. TB Green fluorescence signals were collected during the 60 °C extension step, and CT values were recorded.

### 2.7 Chromatin immunoprecipitation and quantitative PCR (ChIP-qPCR)

To directly verify the transcriptional regulation of FOXD1 by YAP and CD47 by C-MYC, this study employed ChIP-qPCR. A549 cells from each treatment group were cultured to 80% confluence and cross-linked with 1% formaldehyde at room temperature for 10 min, followed by addition of glycine to terminate cross-linking. Cells were washed twice with pre-chilled PBS and lysed on ice for 30 min in SDS lysis buffer containing protease inhibitor cocktail. Chromatin was fragmented to 200–500 bp using a sonicator (Bioruptor Pico, Diagenode). 100 μg of fragmented chromatin was incubated with 2 μg of anti-YAP antibody, anti-C-MYC antibody, or isotype control IgG at 4 °C with rotation overnight. 30 μL of Protein A/G magnetic beads (Thermo Scientific, 88,802) was added and incubation continued at 4 °C for 4 h. The bead-antibody-protein-DNA complexes were collected using a magnetic stand and washed sequentially with low-salt, high-salt, LiCl, and TE buffers, and finally eluted with elution buffer containing 1% SDS, followed by cross-link reversal at 65 °C overnight. DNA was recovered using a DNA purification kit (Qiagen, 28,104) and enrichment at the FOXD1 and CD47 promoter regions was detected by qPCR. Primer sequences were as follows: FOXD1 promoter: forward 5’-GCCGCTGTGTGTTCTGTGAG-3′, reverse 5’-GCTGCCGCGAGTTCTGTCT-3′; CD47 promoter: forward 5’-CGCCGCCCCTCTCTACTTAA-3′, reverse 5’-CGGGATTTAG GGTGGGTTCT-3′; negative control primers were also included. Relative enrichment was calculated using the 2^-ΔΔCt method and normalized to Input DNA as internal control. All ChIP-qPCR experiments were performed with three independent biological replicates.

### 2.8 Flow cytometry for apoptosis detection

After digestion and treatment, A549 cells were gently pipetted and collected, then centrifuged. The supernatant was removed, cells were washed once with PBS, resuspended, and counted. 1 × 10^5^ A549 cells were taken, centrifuged to remove supernatant, and 195 μL of Annexin V/FITC and 10 μL of propidium iodide (PI) staining solution were added, mixed, and incubated at room temperature in the dark for 15 min. Detection was immediately performed using a flow cytometer.

### 2.9 Flow cytometry for cell cycle analysis

After treatment, A549 cells were washed twice with PBS to remove residual medium. After digestion, A549 cells were resuspended in 1 mL of pre-chilled PBS and transferred to 1.5 mL centrifuge tubes. Cells were centrifuged again and the supernatant was aspirated. 1 mL of pre-chilled 75% ethanol was added to the cell pellet, gently pipetted to mix, and fixed at 4 °C overnight. The next day, cells were centrifuged, supernatant discarded, and washed three times with PBS to remove residual ethanol. 0.5 mL of PI was added to each cell sample, mixed, and incubated at 37 °C for 30 min. Subsequently, cell cycle was determined by flow cytometry.

### 2.10 Plate colony formation assay

After treatment, A549 cell medium was discarded, cells were washed with PBS to remove residual medium, digested, centrifuged, resuspended, and counted. A549 cells were plated at 800 cells/well in 6-well plates and cultured for 14 days. Medium was discarded and cells were washed with PBS to remove residual medium. 1 mL of 4% paraformaldehyde was added to each well and fixed at room temperature for 20 min. Paraformaldehyde solution was discarded, cells were washed twice with PBS, and 2 mL of crystal violet staining solution was added to each well and incubated at room temperature for 30 min. Staining solution was discarded and cells were washed with tap water to remove residual stain, then air-dried and photographed.

### 2.11 Transwell assay

Matrigel was thawed on ice and diluted with serum-free medium (1:4). 100 μL of Matrigel was added to the Transwell upper chamber and incubated at 37 °C for 1 h to allow complete solidification. After treatment, A549 cell medium was discarded and cells were washed with PBS to remove residual medium. After digestion and centrifugation, cells were resuspended in serum-free medium to 1 × 10^5^/mL. 200 μL of cell suspension was added to the Transwell upper chamber, and 800 μL of medium containing 10% FBS was added to the lower chamber, followed by incubation for 48 h. Cells and Matrigel in the Transwell were removed, medium discarded, washed twice with PBS, and a cotton swab was used to gently wipe off non-migrated cells and Matrigel on the upper surface. Transwells were immersed in 24-well plates containing 4% methanol for fixation for 20 min. PBS washing was performed twice. 0.1% crystal violet staining solution was added and stained at room temperature for 20 min. PBS washing was performed three times. After air-drying, observation and photography were performed under an optical microscope, and the number of invasive cells was counted.

### 2.12 Scratch assay

A549 cells were seeded at 1 × 10^5^ cells/well in 6-well plates and cultured until complete confluence, then scratched uniformly in the same direction using a 200 μL pipette tip, controlling scratch width at approximately 50 μm. Cells were washed 1–2 times with PBS and then replaced with serum-free medium. Imaging was performed at the same position at 0 h and 48 h, and cell images were captured using an inverted microscope to observe changes in scratch width.

### 2.13 Statistical analysis

Experimental data are presented as mean ± standard deviation. Comparisons among multiple groups were performed using one-way analysis of variance (ANOVA), followed by Tukey’s HSD (or Dunnett’s) post-hoc test to correct for multiple comparisons. *p* < 0.05 was considered statistically significant. All analyses were performed using GraphPad Prism 9 software.

## 3 Results

### 3.1 Bulk RNA-seq analysis

**Figure 1:**
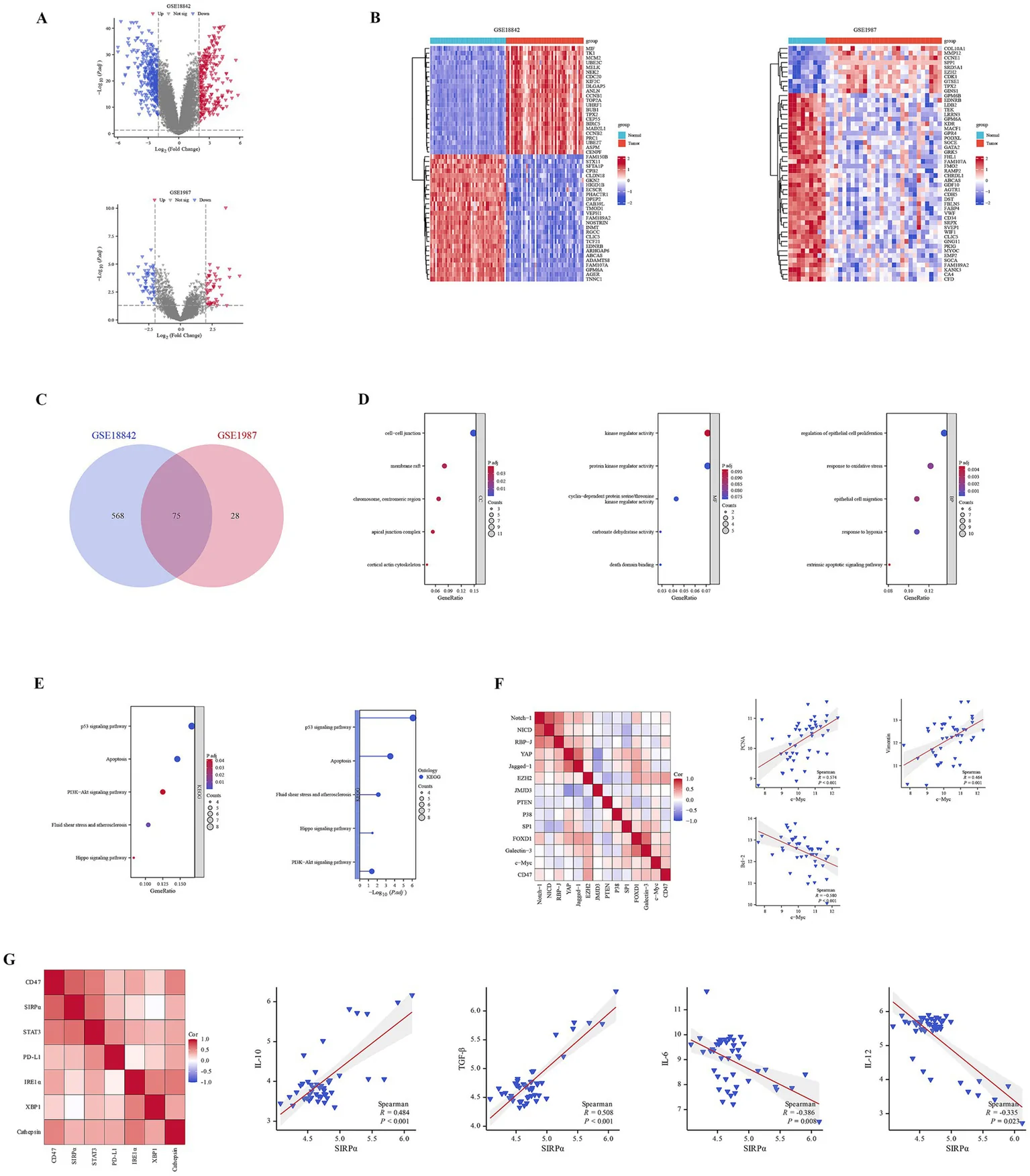
Bulk RNA-seq analysis. **(A)** Volcano plots of differentially expressed genes in GSE1987/GSE18842. **(B)** Heatmaps of differentially expressed genes in GSE1987/GSE18842. **(C)** Venn diagram of differentially expressed genes in GSE1987/GSE18842. **(D)** Cellular component (CC) enrichment bubble plot, molecular function (MF) enrichment bubble plot, and biological process (BP) enrichment bubble plot. **(E)** KEGG pathway enrichment bubble plot and KEGG pathway enrichment lollipop plot. **(F)** Correlation heatmap of 14 factors including Notch1, NICD, RBP-J; scatter plot of C-MYC correlation with proliferation marker PCNA; scatter plot of C-MYC correlation with invasion marker Vimentin; scatter plot of C-MYC correlation with apoptosis marker Bcl-2. **(G)** Correlation matrix heatmap of immune checkpoint molecules (CD47/SIRP*α*/PD-L1) with endoplasmic reticulum stress pathway (IRE1α/XBP1/Cathepsin) and STAT3 signaling; Scatter plot of SIRPα expression correlation with M2-type macrophage polarization markers (IL-10/TGF-*β*); Scatter plot of SIRPα expression correlation with M1-type macrophage polarization markers (IL-6/IL-12).

This study integrated the GSE1987 (103 differential genes identified, 48 upregulated/55 downregulated) and GSE18842 (643 differential genes identified, 270 upregulated/373 downregulated) datasets, yielding 75 core intersection genes through Venny analysis. Further functional enrichment analysis revealed that these genes were significantly enriched in cell–cell junctions, membrane rafts, and chromosomal centromeric regions; at the molecular function level, they primarily involved kinase regulatory activity, protein kinase regulatory activity, and cyclin-dependent protein serine/threonine kinase regulatory activity; at the biological process level, they focused on regulation of epithelial cell proliferation, oxidative stress response, and epithelial cell migration. KEGG analysis revealed significant enrichment of these genes in key oncogenic pathways such as the p53 signaling pathway, apoptosis, fluid shear stress and atherosclerosis, Hippo signaling pathway, and PI3K-Akt signaling pathway. Spearman correlation networks revealed multi-dimensional regulatory mechanisms: NOTCH1 showed strong positive correlation with the intracellular domain NICD, NICD positively correlated with the transcription factor RBP-J, and the ligand JAGGED1 synergistically enhanced NOTCH1; YAP activated EZH2 expression and inhibited JMJD3, with EZH2 and JMJD3 negatively correlated; EZH2 inhibited PTEN while JMJD3 promoted PTEN; p38 positively correlated with the transcription factor SP1, which negatively regulated NOTCH1; YAP drove FOXD1 transcription, FOXD1 promoted Galectin-3, Galectin-3 activated C-MYC, and C-MYC positively regulated CD47, PCNA (proliferation marker), Vimentin (invasion marker), and inhibited Bcl-2 (apoptosis marker) (Figure 1).

### 3.2 Identification, characterization, and functional analysis of malignant cell subpopulations

**Figure 2:**
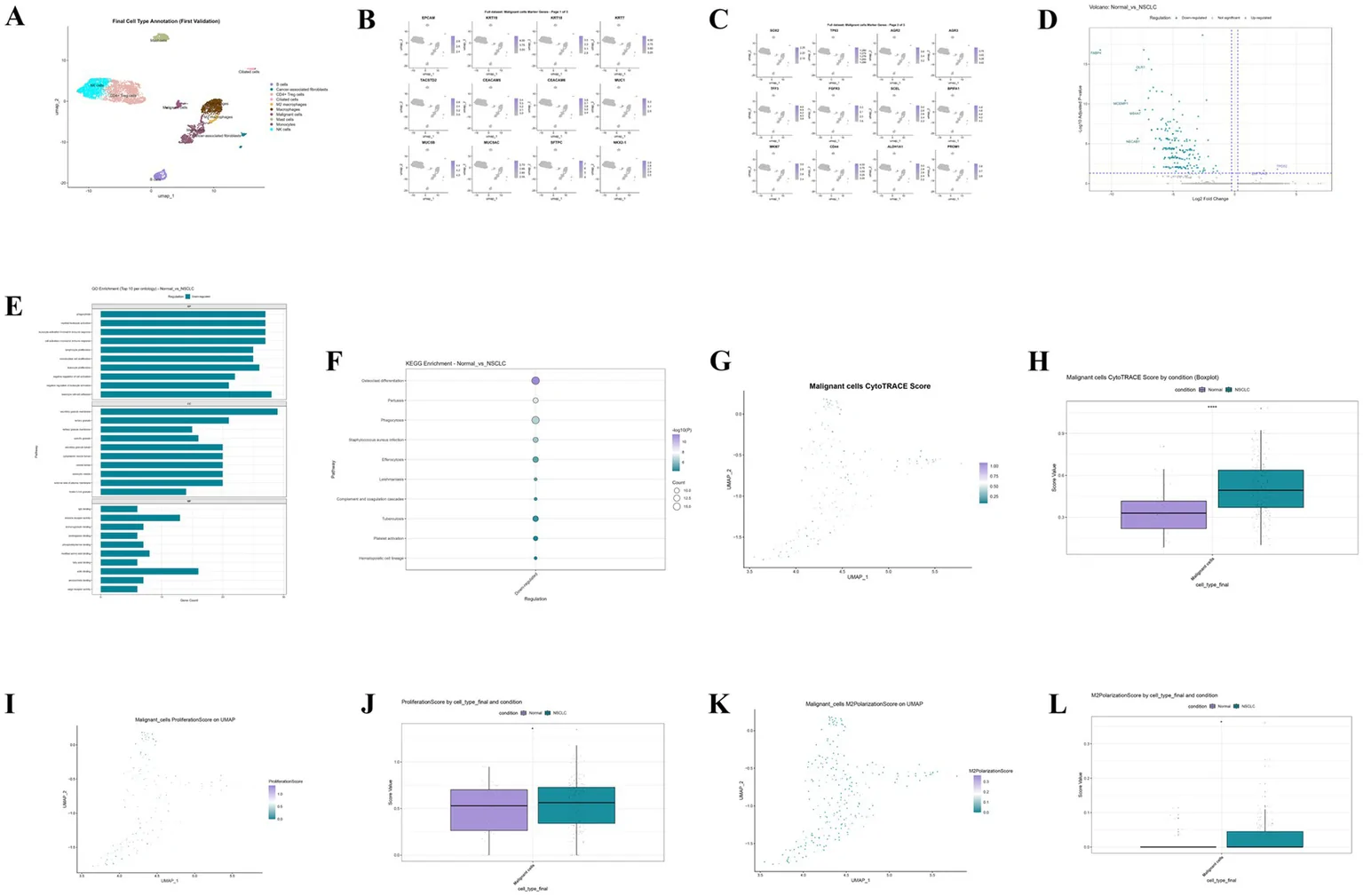
Identification, characterization, and functional analysis of malignant cell subpopulations. **(A)** UMAP clustering visualization of single-cell transcriptome data. Different colors represent cell types annotated by specific marker genes. **(B,C)** Feature gene expression distribution plots of malignant cells. Color intensity represents gene expression levels (Log normalized counts), with darker purple indicating higher expression and gray indicating undetected or low expression. **(D)** Volcano plot of differentially expressed genes between normal tissues and NSCLC. X-axis represents Log2 Fold Change, Y-axis represents -Log10 Adjusted *p*-value. Cyan dots represent significantly downregulated genes, purple dots represent significantly upregulated genes, gray dots represent genes with no significant difference. Key differentially expressed genes (such as FABP4, TPD52, etc.) are labeled. **(E)** GO functional enrichment analysis bar chart. Showing the top 10 significantly enriched terms for downregulated genes in normal tissue versus NSCLC comparison. Divided into biological process (BP), cellular component (CC), and molecular function (MF) categories, with X-axis representing gene count, bar length representing the number of enriched genes. **(F)** KEGG pathway enrichment analysis bubble plot. Showing significantly enriched KEGG signaling pathways for downregulated genes. Y-axis represents pathway names, bubble size represents gene count enriched in the pathway (Count), bubble color intensity represents significance level (−log10(*P*)), with darker color indicating smaller *p* value and more significant enrichment. **(G)** UMAP distribution of malignant cell stemness scores. Showing the distribution of stemness scores (CytoTRACE Score) in UMAP space within the malignant cell subpopulation, with color intensity representing score levels. **(H)** Box plot of malignant cell stemness scores. Comparing differences in stemness scores (CytoTRACE Score) of malignant cells between normal tissues and NSCLC samples. **(I)** UMAP distribution of malignant cell proliferation scores. Showing the distribution of proliferation scores (ProliferationScore) in UMAP space within the malignant cell subpopulation. **(J)** Box plot of malignant cell proliferation scores. Comparing differences in proliferation scores of malignant cells between normal tissues and NSCLC samples. **(K)** UMAP distribution of malignant cell M2 polarization scores. Showing the distribution of M2 polarization scores (M2PolarizationScore) in UMAP space within the malignant cell subpopulation. **(L)** Box plot of malignant cell M2 polarization scores. Comparing M2 polarization scores of malignant cells between normal tissues and NSCLC samples. Asterisks above box plots indicate statistically significant differences between groups, *p < 0.05, ****p < 0.0001.

To deeply investigate the tumor microenvironment heterogeneity of non-small cell lung cancer (NSCLC), dimensionality reduction and clustering analysis were performed on single-cell transcriptome data. The UMAP clustering plot displays the final cell type annotation results. Based on the expression patterns of specific marker genes, cell populations were successfully divided into multiple independent subpopulations including B cells, cancer-associated fibroblasts, CD4+Treg cells, ciliated cells, M2 macrophages, macrophages, mast cells, monocytes, NK cells, and malignant cells; among these, malignant cells were clearly identified and separated as an independent cell cluster, laying the foundation for subsequent targeted analyses (Figure 2A). To confirm the malignant nature of this subpopulation, a series of feature gene expression distribution plots display the transcriptional levels of malignant cell-specific marker genes. Results showed that epithelial cell adhesion molecule (EPCAM), keratin family members (KRT7, KRT18, KRT19), and carcinoembryonic antigen-related genes (CEACAM5, CEACAM6) exhibited significantly high expression in the malignant cell cluster; simultaneously, the lung adenocarcinoma-associated transcription factor NKX2-1 and surfactant protein SFTPC were also mainly enriched in this subpopulation, strictly validating cell annotation accuracy at the transcriptional level (Figures 2B, C). On this basis, through differential expression analysis comparing normal tissues and NSCLC samples, the volcano plot visually presented the significantly differentially expressed genes identified, among which FABP4, OLR1, MCEMP1 and other genes showed significant downregulation in NSCLC (Log2 Fold Change < 0 with significant *p* values), while TPD52 and other genes showed significant upregulation (Log2 Fold Change > 0 with significant *p* values, Figure 2D). Functional enrichment analysis further revealed the biological significance of these differential genes. The GO enrichment bar chart showed that downregulated genes were mainly significantly enriched in biological processes (BP) such as phagocytosis, myeloid leukocyte activation, and immune response; cellular components (CC) such as secretory granule membrane; and molecular functions (MF) such as immune receptor activity (Figure 2E). Furthermore, KEGG pathway enrichment analysis bubble plots indicated that differential genes were mainly involved in osteoclast differentiation, phagosome, *Staphylococcus aureus* infection, and tuberculosis signaling pathways, suggesting that NSCLC development is accompanied by significant immune microenvironment remodeling and specific metabolic pathway alterations (Figure 2F). To explore the biological characteristics of malignant cells, stemness, proliferation, and M2 polarization feature scores were analyzed for the malignant cell subpopulation. UMAP dimensionality reduction plots showed that stemness scores (CytoTRACE Score) of malignant cells exhibited heterogeneous distribution across cell subpopulations, with some cell regions showing higher stemness features (Figure 2G). Box plot analysis showed that compared with normal tissues, stemness scores of malignant cells in NSCLC samples were significantly increased (*p* < 0.0001, Figure 2H). Regarding proliferation features, UMAP plots showed that proliferation scores (ProliferationScore) were unevenly distributed in malignant cells, with some cell subpopulations having higher proliferative activity (Figure 2I). Box plot results showed that proliferation scores of malignant cells in NSCLC samples were significantly higher than those in normal tissues (*p* < 0.05, Figure 2J). Additionally, UMAP distribution of M2 polarization scores (M2PolarizationScore) showed that there were cell subpopulations with M2-type macrophage polarization features within malignant cells (Figure 2K). Box plot analysis showed that M2 polarization scores of malignant cells in NSCLC samples were significantly higher than those in normal tissues (*p* < 0.05, Figure 2L). The above results indicate that malignant cell subpopulations in NSCLC have higher stemness, proliferative activity, and M2-like polarization features, suggesting they may possess stronger malignant potential, laying a foundation for further functional studies.

### 3.3 Developmental trajectories, cell communication, and key regulatory mechanisms of malignant cells

**Figure 3:**
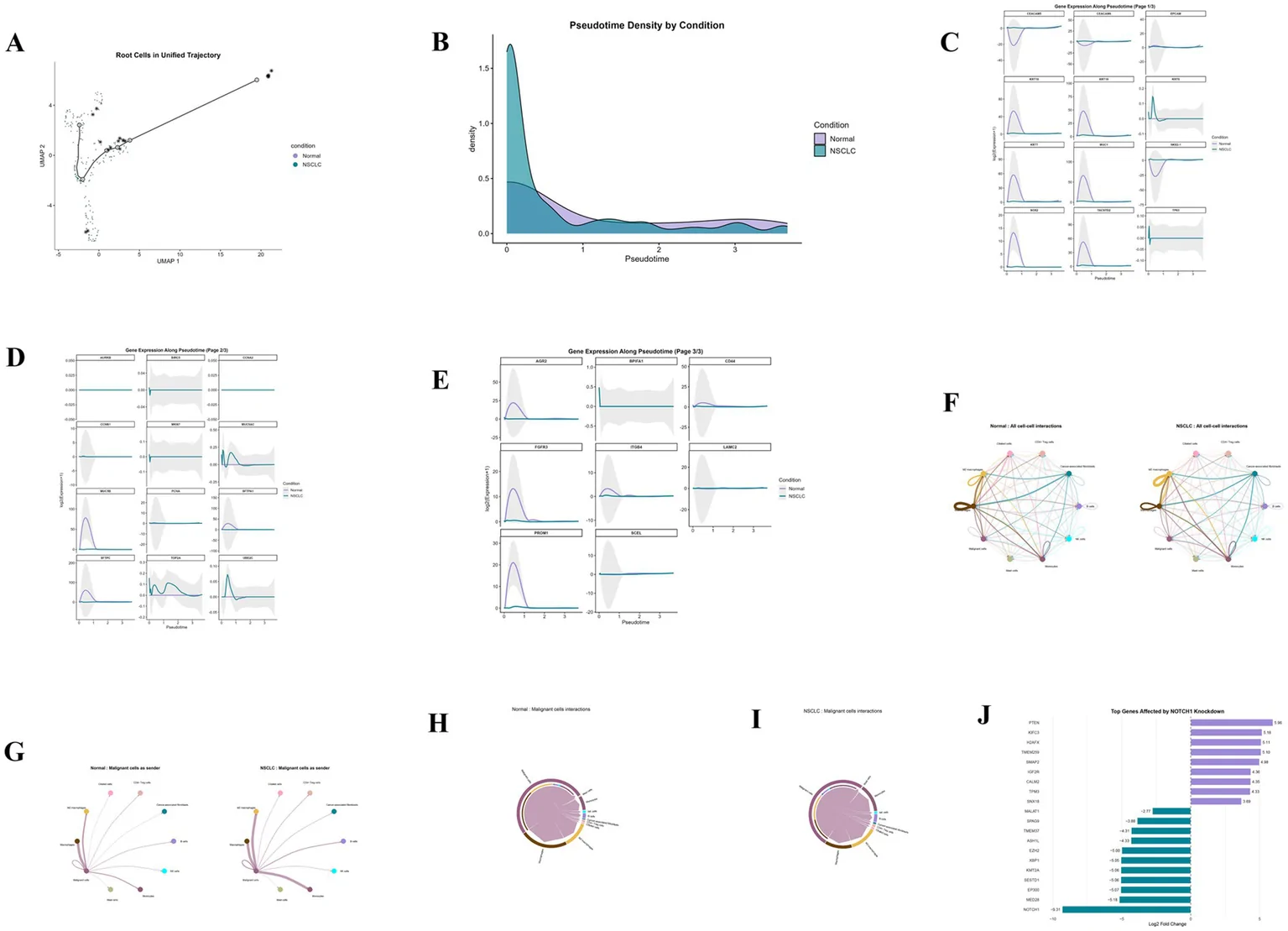
Developmental trajectories, cell communication, and key regulatory mechanisms of malignant cells. **(A)** UMAP distribution of root cells in unified trajectory. Showing the distribution of malignant cells from Normal and NSCLC samples on the unified developmental trajectory, with asterisks marking inferred root cell positions and numbered circles indicating key branch nodes. **(B)** Pseudotime density distribution plots by group. Showing probability density distribution differences of Normal and NSCLC cells along the pseudotime axis, reflecting the enrichment of cells at different stages of the developmental trajectory. **(C–E)** Gene expression dynamics of key genes along pseudotime. Showing expression trends (log2-transformed values) of epithelial markers, proliferation-related genes, and stem cell markers in Normal and NSCLC cells along pseudotime progression, with gray shaded areas representing confidence intervals. **(F)** Comparison of global cell interaction networks between Normal and NSCLC. Showing communication network topologies among all cell subpopulations in both conditions, with line thickness representing interaction intensity. **(G)** Interaction networks with malignant cells as signal senders in Normal and NSCLC. Specifically showing communication network differences with malignant cells as the source sending signals to other cell subpopulations. **(H,I)** Chord diagrams of malignant cell interactions in Normal and NSCLC. Quantitatively showing bidirectional interaction weights and flow directionality between malignant cells and other specific cell subpopulations (such as macrophages, M2 macrophages), with sector size and connection width corresponding to interaction weights. **(J)** Changes in key gene expression after virtual NOTCH1 knockout. Showing the response changes in expression levels of related genes after simulated NOTCH1 knockout in the malignant cell subpopulation.

Based on the high stemness and proliferative activity exhibited by malignant cell subpopulations, to further analyze their developmental trajectories and microenvironment interaction mechanisms, this study constructed pseudotime analysis and performed cell communication network inference. The unified trajectory UMAP projection plot showed that Normal and NSCLC-derived malignant cells were distributed along the same main trajectory, with Root Cells mainly localized in the NSCLC sample aggregation region, suggesting that tumor cells may serve as the initiating or driving state of this differentiation trajectory (Figure 3A). Pseudotime density distribution plots further confirmed that NSCLC cells were significantly enriched in the early pseudotime stage, while Normal cells were mainly distributed in the middle and late stages, indicating a significant temporal offset in developmental progression between the two groups (Figure 3B). Gene expression dynamics analysis along pseudotime revealed that the transcriptional profiles of this subpopulation showed significant intergroup heterogeneity with pseudotime changes: in the early pseudotime stage (0–1 interval), expression of epithelial and secretion-related genes in normal cells (including KRT18, KRT19, KRT7, MUC1, SOX2, TACSTD2, and MUC5B, SFTPA1, SFTPC) showed significant upregulation with distinct peaks, while AGR2, FGFR3, and PROM1 also showed similar early high-expression peaks in the normal group. These high-expression features of normal cells were largely absent in NSCLC samples, with corresponding curves mostly remaining in a low-level state. In contrast, NSCLC subpopulations exhibited their unique transcriptional dynamics, such as significant fluctuations and upregulation of cell proliferation-related genes (CCNB1, TOP2A, UBE2C) and MUC5AC in early pseudotime, while such activation intensity was not observed in normal cells. Furthermore, for some of the analyzed genes (such as CEACAM family, AURKB, BIRC5, CCNA2, PCNA, SCEL, LAMC2, etc.), expression in both groups remained relatively stable along pseudotime evolution, with relatively small intergroup differences. The above results suggest that the transcriptional regulatory mechanisms of the malignant cell subpopulations identified by clustering differ significantly from normal cell differentiation processes along dynamic developmental trajectories, and exhibit specific cancer-related expression features (Figures 3C–E). In addition, cell communication network analysis displayed the interaction profiles of malignant cells under different states. The global interaction network plot showed that the connection density and intensity among cell subpopulations within the NSCLC group were altered compared with the Normal group, with malignant cells forming tighter connections with other stromal and immune cells (Figure 3F). Directional interaction plots with malignant cells as signal senders showed that in the NSCLC microenvironment, signal flow from malignant cells to monocytes, M2 macrophages, and cancer-associated fibroblasts was significantly enhanced (Figure 3G). Chord diagram quantitative visualization further confirmed that the interaction weights between malignant cells and macrophages/M2 macrophages in the NSCLC group were significantly higher than in the Normal group, with specific interaction flow directionality (Figures 3H,I). The above results reveal the abnormal developmental trajectory arrest of NSCLC malignant cells and their active regulatory role in remodeling the immune microenvironment. To further explore key regulatory factors driving the above malignant phenotypes, this study used virtual knockout technology to evaluate the regulatory effects of key signaling molecules on downstream gene expression. Gene expression response analysis plots showed that after simulated NOTCH1 gene knockout in the malignant cell subpopulation, the expression level of the tumor suppressor gene PTEN showed a significant upward trend; in contrast, other tested genes or control groups did not show comparable specific changes. This result intuitively reveals that in this NSCLC malignant cell subpopulation, the NOTCH1 signaling pathway exerts a significant negative regulatory effect on PTEN, suggesting that high NOTCH1 expression may be an important upstream mechanism leading to PTEN functional loss and malignant tumor progression (Figure 3J).

### 3.4 Notch1 promotes NSCLC tumorigenesis through regulation of the FOXD1/galectin-3/CD47 pathway

**Figure 4:**
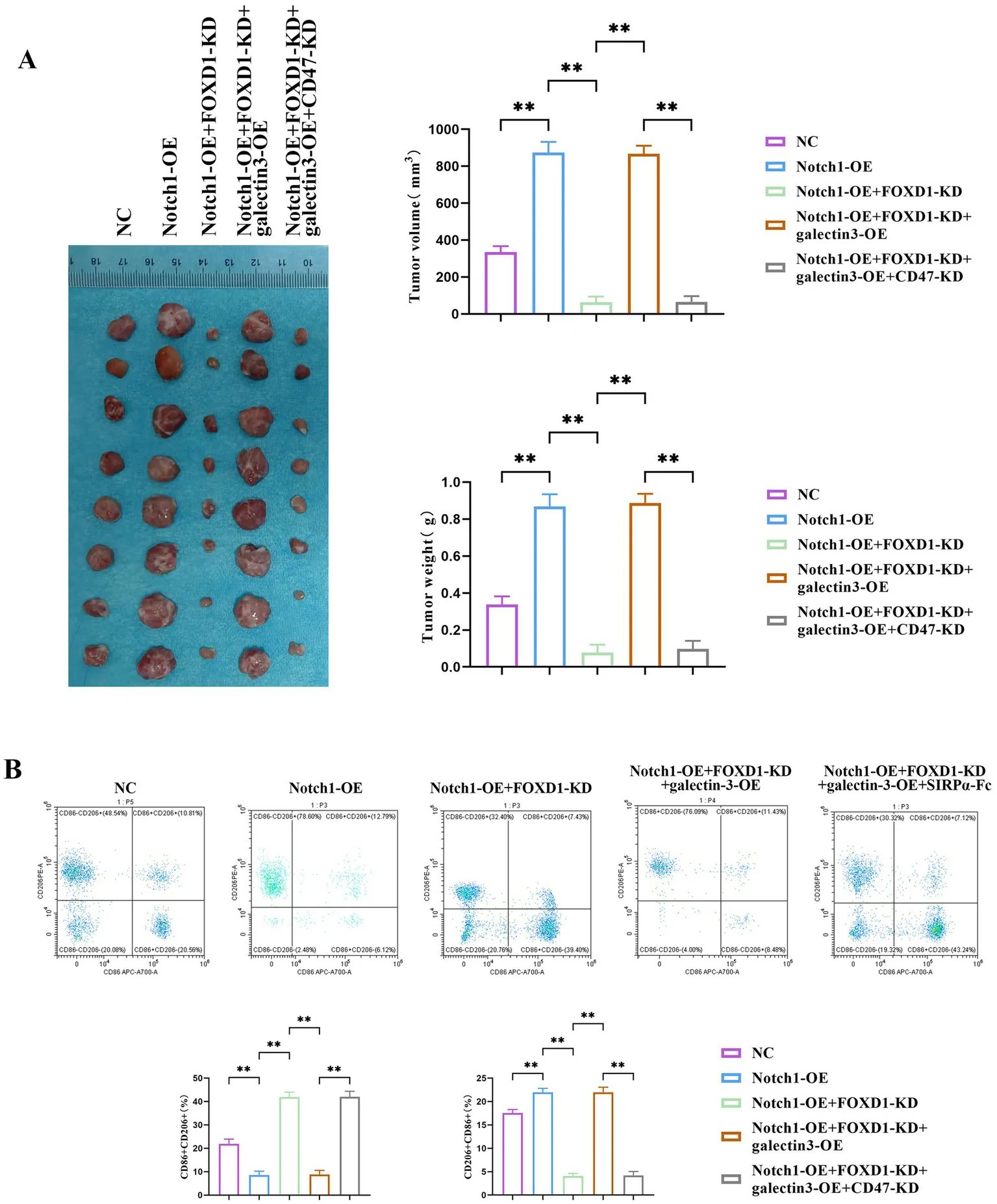
Notch1 promotes NSCLC tumorigenesis through regulation of the FOXD1/galectin-3/CD47 pathway. **(A)** Nude mouse subcutaneous xenograft tumor results and statistical analysis of tumor weight and volume. Data are presented as mean ± standard deviation. **(B)** Flow cytometry analysis and statistical quantification of CD86^+^ M1-macrophages and CD206^+^ M2-macrophages within F4/80^+^ TAMs from tumor tissues. *N* = 3, *p* < 0.05 was considered statistically significant, ns *p* > 0.05, *p < 0.05, **p < 0.01.

The *in vivo* experimental grouping and treatment procedures are detailed in Supplementary Table S1. To investigate the role of Notch1 in NSCLC and its molecular mechanisms, a nude mouse subcutaneous tumorigenesis experiment was performed. Nude mouse subcutaneous tumorigenesis results showed that tumor weight and volume in the Notch1-OE group were higher than those in the NC group. Compared with the Notch1-OE group, tumor weight and volume in the Notch1-OE+FOXD1-KD group were significantly reduced. Compared with the Notch1-OE+FOXD1-KD group, tumor weight and volume in the Notch1-OE+FOXD1-KD+galectin-3-OE group were significantly increased. Compared with the Notch1-OE+FOXD1-KD+galectin-3-OE group, tumor weight and volume in the Notch1-OE+FOXD1-KD+galectin-3-OE+CD47-KD group were significantly reduced (Figure 4A). These results indicate that Notch1 promotes NSCLC tumorigenesis through regulation of the FOXD1/galectin-3/CD47 pathway.

To further evaluate the impact of the Notch1 signaling axis on TAM polarization in the tumor microenvironment, flow cytometry analysis was performed on subcutaneous xenograft tumor tissues. In the NC group, total macrophages (F4/80^+^) accounted for 15% of live cells, with no significant differences across groups, indicating that different genetic manipulations did not alter total macrophage infiltration numbers. However, macrophage polarization states changed significantly among groups. Within the F4/80^+^ gate, the proportion of M2-type marker CD206-positive cells in the NC group was 50%, consistent with tumor volume trends. The proportion of M1-type marker CD86-positive cells was 20%, with trends opposite to tumor volume. These results indicate that Notch1 drives TAM polarization toward the M2 phenotype *in vivo* through the FOXD1/galectin-3/CD47 axis, without altering total macrophage infiltration numbers. The M2/M1 ratio positively correlated with tumor growth trends (Figure 4B).

### 3.5 Notch1 regulates nuclear YAP levels through the YAP/EZH2/PTEN axis

**Figure 5:**
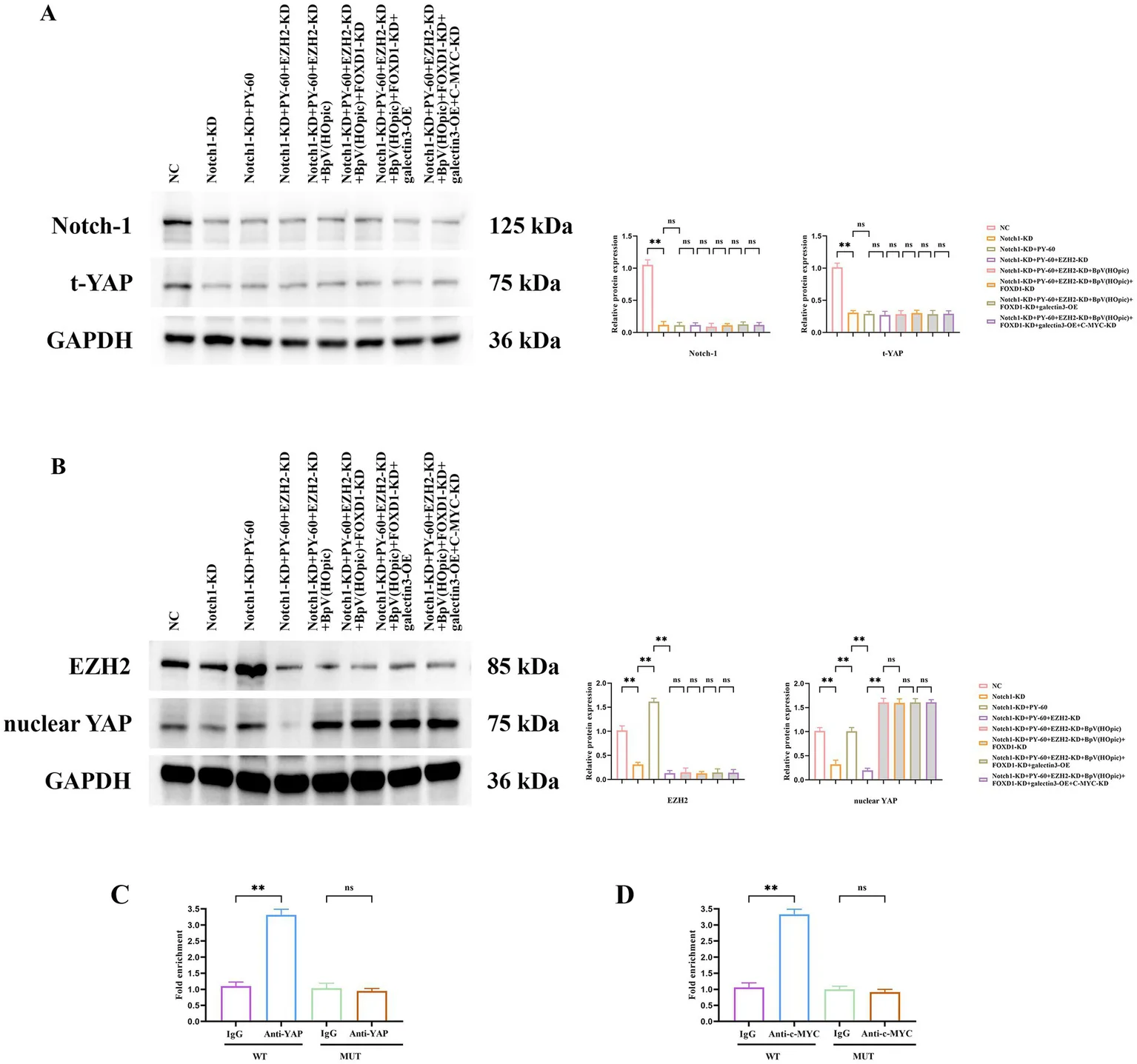
Notch1 promotes NSCLC through regulation of the YAP/EZH2/JMJD3/PTEN/FOXD1/galectin-3/CD47 signaling pathway. **(A)** Western blot detection of Notch1 and t-YAP bands in co-cultured A549 cells and statistical analysis of relative protein expression levels. **(B)** Western blot detection of EZH2 and nuclear YAP bands in co-cultured A549 cells and statistical analysis of relative protein expression levels. **(C)** ChIP-qPCR analysis of YAP enrichment on the FOXD1 promoter region. The data are presented as a percentage of the input DNA (mean ± SD, *n* = 3). **(D)** ChIP-qPCR analysis of C-MYC enrichment on the CD47 promoter region. The data are presented as a percentage of the input DNA (mean ± SD, *n* = 3).

To elucidate the molecular mechanism by which Notch1 regulates FOXD1/galectin-3/CD47 signaling, Western blot analysis was performed to examine the expression of related proteins in A549 cells. Western blot results showed that Notch1 knockdown significantly reduced total YAP, EZH2, and nuclear YAP levels, while addition of the YAP agonist PY-60 rescued EZH2 and nuclear YAP expression, but did not affect Notch1 and total YAP levels, suggesting that YAP is downstream of Notch1 and participates in positive feedback regulation. Further knockdown of EZH2 eliminated PY-60-induced nuclear YAP elevation, while PTEN inhibition (BpV(HOpic)) restored nuclear YAP levels, indicating that EZH2-mediated PTEN suppression is a key step in YAP nuclear translocation. In contrast, FOXD1 knockdown, galectin-3 overexpression, or C-MYC knockdown did not alter Notch1, total YAP, EZH2, and nuclear YAP expression, indicating that these molecules act downstream of this pathway and do not participate in feedback regulation of YAP activation status. The complete dataset is shown in (Figure 5). These results indicate that Notch1 regulates YAP nuclear translocation through the YAP/EZH2/PTEN axis, while FOXD1, galectin-3, and C-MYC are downstream effector molecules of this signaling axis and do not participate in feedback regulation of YAP activation status.

To verify the transcriptional regulation of FOXD1 by YAP and CD47 by C-MYC, ChIP-qPCR experiments were performed. Results showed that in the Notch1-OE group, YAP enrichment at the FOXD1 promoter region was significantly higher than in the NC group, and this enrichment was blocked by Verteporfin treatment (Figure 5D), indicating that YAP can directly bind to the FOXD1 promoter and activate its transcription. Meanwhile, compared with the NC group, C-MYC enrichment at the CD47 promoter region was also significantly increased in the Notch1-OE group, and this effect was also eliminated by Verteporfin (Figure 5C), confirming that C-MYC can directly bind to the CD47 promoter and drive its expression. The above results provide direct molecular evidence at the protein-DNA interaction level for the two key transcriptional regulatory relationships of “YAP/FOXD1” and “C-MYC/CD47.”

### 3.6 Notch1 drives CD47 expression and M2 polarization through the YAP/EZH2/PTEN/FOXD1/galectin-3/C-MYC axis

**Figure 6:**
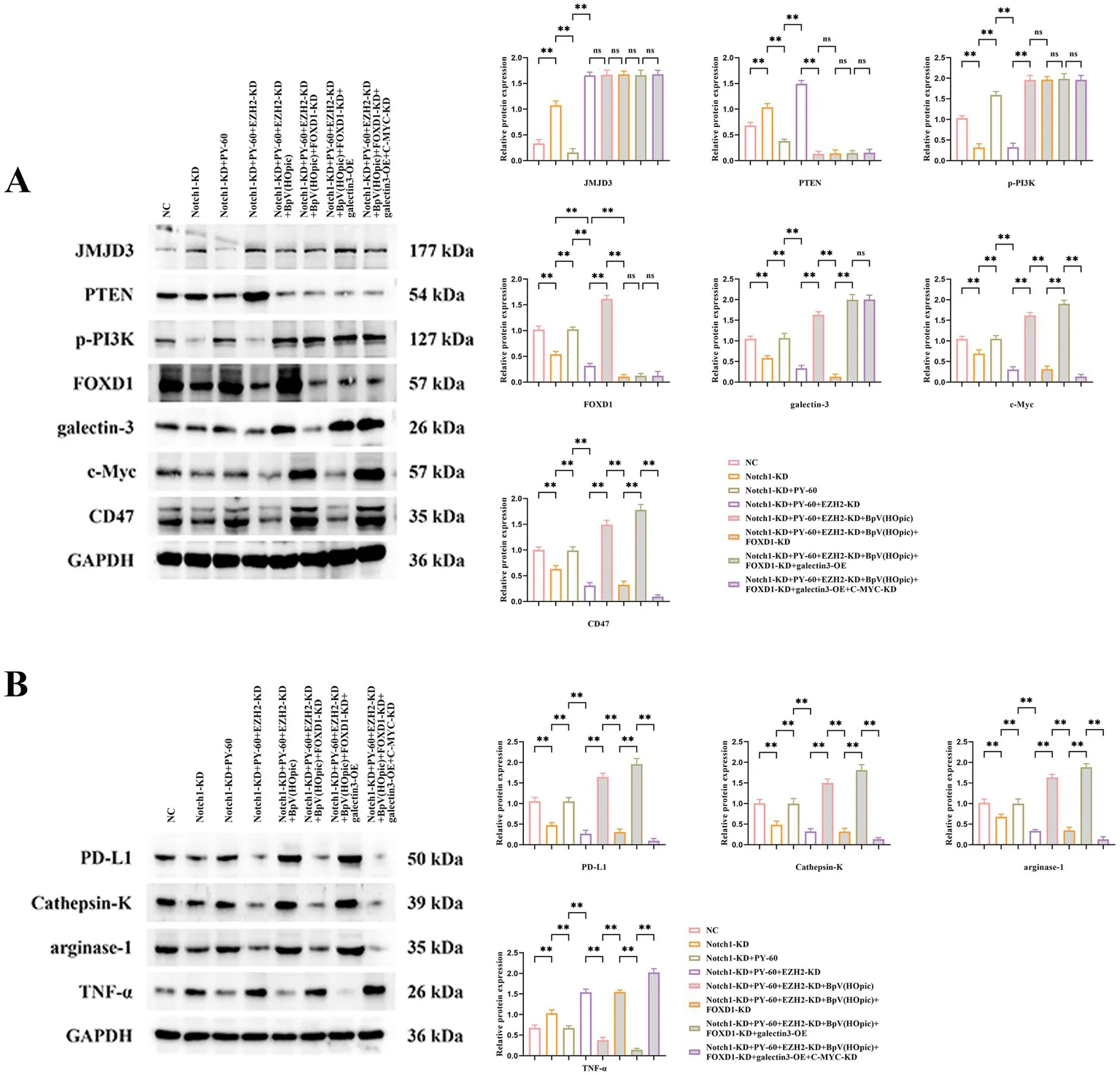
Notch1 induces TAM M2 polarization through the YAP/EZH2/PTEN/FOXD1/galectin-3/CD47 signaling axis. **(A)** Western blot analysis of JMJD3, PTEN, p-PI3K, FOXD1, galectin-3, C-MYC and CD47 protein levels in co-cultured A549 cells, followed by quantitative analysis of relative protein expression. GAPDH was used as the loading control. **(B)** Western blot examination of PD-L1, Cathepsin-K, arginase-1 and TNF-α associated with M2-polarization in co-cultured THP-1-derived macrophages, with quantification of relative protein abundance. All data are shown as mean ± SD, *n* = 3. ns, no significance; *p < 0.05, **p < 0.01.

To analyze how Notch1 drives CD47 expression and M2 polarization through the YAP/EZH2/PTEN/FOXD1/galectin-3/C-MYC axis, Western blot and qRT-PCR were used to detect protein bands and perform statistical analysis of relative protein expression levels in co-cultured cells. Western blot results showed that compared with the NC group, expression levels of p-PI3K, FOXD1, galectin-3, C-MYC, and CD47 in the Notch1-KD group were significantly reduced, while JMJD3 and PTEN expression levels were significantly increased. Compared with the Notch1-KD group, JMJD3 and PTEN expression levels in the Notch1-KD+PY-60 group showed no significant change, while p-PI3K, FOXD1, galectin-3, C-MYC, and CD47 expression levels were significantly increased. Compared with the Notch1-KD+PY-60 group, p-PI3K, FOXD1, galectin-3, C-MYC, and CD47 expression levels in the Notch1-KD+PY-60+EZH2-KD group were decreased, while JMJD3 and PTEN expression levels were increased. Compared with the Notch1-KD+PY-60+EZH2-KD group, EZH2 and JMJD3 expression levels in the Notch1-KD+PY-60+EZH2-KD+BpV(HOpic) group showed no significant difference, p-PI3K, FOXD1, galectin-3, and C-MYC expression levels were increased, CD47 expression levels recovered, and PTEN expression levels were significantly decreased. Compared with the Notch1-KD+PY-60+EZH2-KD+BpV(HOpic) group, JMJD3, PTEN, p-PI3K, FOXD1, and CD47 expression levels in the Notch1-KD+PY-60+EZH2-KD+BpV(HOpic)+FOXD1-KD group showed no significant change, while FOXD1, galectin-3, C-MYC, and CD47 expression levels were significantly decreased. Compared with the Notch1-KD+PY-60+EZH2-KD+BpV(HOpic)+FOXD1-KD group, JMJD3, PTEN, p-PI3K, and FOXD1 expression levels in the Notch1-KD+PY-60+EZH2-KD+BpV(HOpic)+FOXD1-KD+galectin-3-OE group showed no significant difference, while galectin-3, C-MYC, and CD47 expression levels significantly recovered. Compared with the Notch1-KD+PY-60+EZH2-KD+BpV(HOpic)+FOXD1-KD+galectin-3-OE group, JMJD3, PTEN, p-PI3K, FOXD1, and galectin-3 protein expression levels in the Notch1-KD+PY-60+EZH2-KD+BpV(HOpic)+ FOXD1-KD+galectin-3-OE+C-MYC-KD group showed no significant difference, while C-MYC and CD47 expression levels were significantly decreased (Figure 6A).

Western blot results showed that compared with the NC group, pD-L1, Cathepsin K, and arginase-1 protein expression levels in the Notch1-KD group were decreased, while TNF-*α* expression levels were increased. Compared with the Notch1-KD group, pD-L1, Cathepsin K, and arginase-1 protein expression levels in the Notch1-KD+PY-60 group were increased, while TNF-*α* expression levels were decreased. Compared with the Notch1-KD+PY-60 group, pD-L1, Cathepsin K, and arginase-1 protein expression levels in the Notch1-KD+PY-60+EZH2-KD group were significantly decreased, while TNF-α expression levels were significantly increased. Compared with the Notch1-KD++PY-60+EZH2-KD group, pD-L1, Cathepsin K, and arginase-1 protein expression levels in the Notch1-KD+PY-60+EZH2-KD+BpV(HOpic) group were increased, while TNF-α expression levels were decreased. Compared with the Notch1-KD+PY-60+EZH2-KD+BpV(HOpic) group, pD-L1, Cathepsin K, and arginase-1 protein expression levels in the Notch1-KD+PY-60+EZH2-KD+BpV (HOpic)+FOXD1-KD group were significantly decreased, while TNF-α expression levels were significantly increased. Compared with the Notch1-KD+PY-60+EZH2-KD+BpV(HOpic)+FOXD1-KD group, pD-L1, Cathepsin K, and arginase-1 protein expression levels in the Notch1-KD+PY-60+EZH2-KD+BpV(HOpic)+FOXD1-KD+galectin-3-OE group significantly recovered again, while TNF-α expression levels were significantly decreased. Compared with the Notch1-KD+PY-60+EZH2-KD+BpV(HOpic)+FOXD1-KD+galectin-3-OE group, pD-L1, Cathepsin K, and arginase-1 protein expression levels in the Notch1-KD+PY-60+EZH2-KD+BpV(HOpic)+FOXD1-KD+galectin-3-OE+C-MYC-KD group were decreased, while TNF-α expression levels were significantly increased (Figure 6B). The above results indicate that Notch1 drives CD47 expression and promotes macrophage M2 polarization through stepwise regulation of the YAP/EZH2/PTEN/FOXD1/galectin-3/C-MYC signaling axis.

### 3.7 CD47 inhibitor blocks Notch1-induced macrophage M2 polarization and STAT3/PD-L1 activation

**Figure 7:**
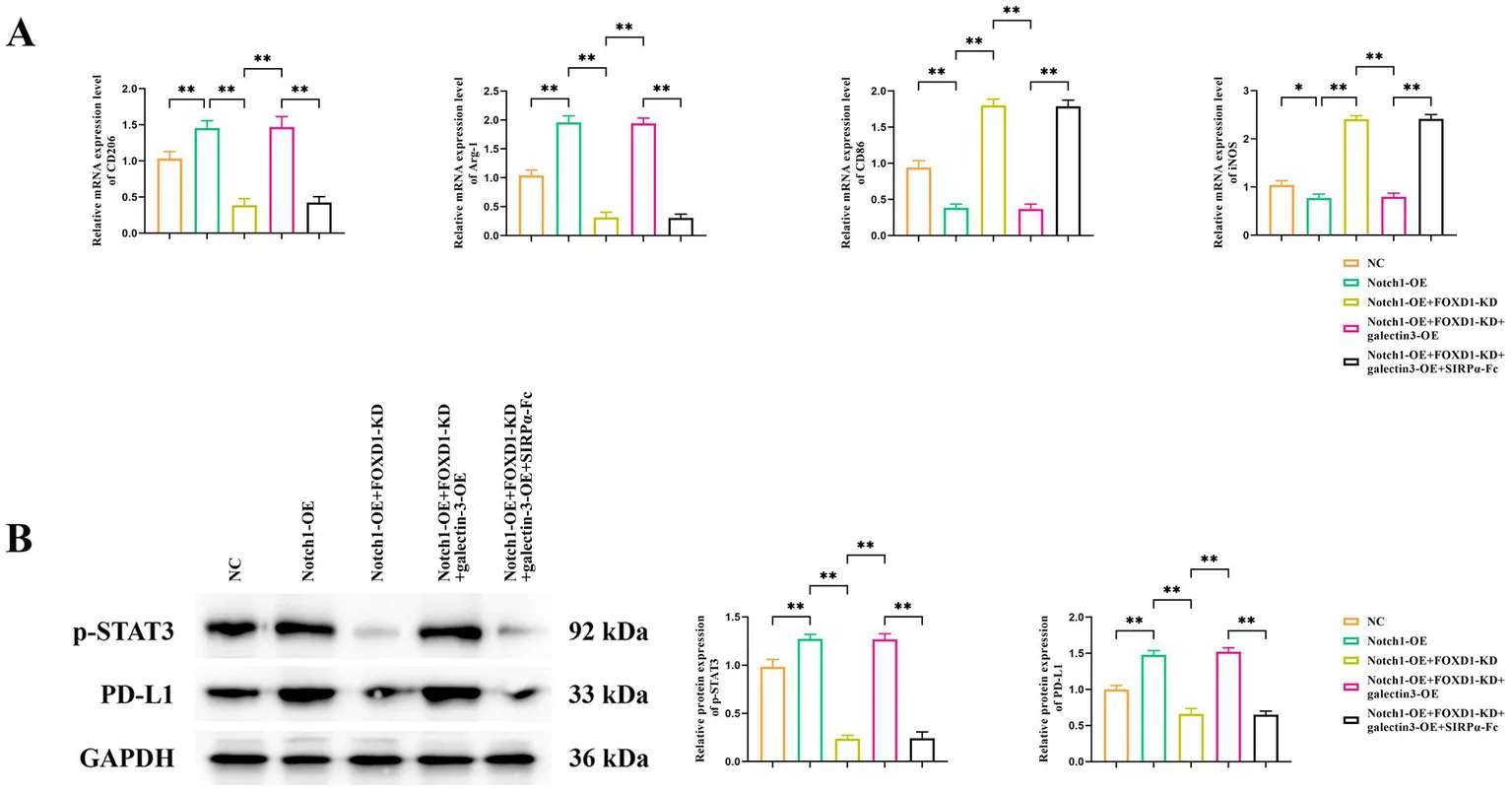
CD47 inhibitor blocks Notch1-induced macrophage M2 polarization and STAT3/PD-L1 activation. **(A)** qRT-PCR detection of relative expression levels of M1 markers (CD86, iNOS) and M2 markers (CD206, Arg-1). **(B)** Western blot detection of representative bands for p-STAT3 and PD-L1. All data are shown as mean ± SD, *n* = 3. *p < 0.05, **p < 0.01.

To verify the functional role of the CD47/SIRPα signaling in Notch1-driven TAM polarization, qRT-PCR was performed to detect M2-type markers: CD206, Arg-1; M1-type markers: CD86, iNOS; and Western blot (detecting THP-1-derived macrophage proteins) for p-STAT3 and PD-L1. qRT-PCR results showed that compared with the NC group, M2 markers CD206 and Arg-1 mRNA levels in macrophages of the Notch1-OE group were increased approximately 4-fold and 5-fold, respectively, while M1 markers CD86 and iNOS were decreased by approximately 60–70%. FOXD1 knockdown reversed these changes, while galectin-3 overexpression restored the M2 phenotype. Notably, addition of CD47 neutralizing antibody significantly inhibited Notch1-OE+FOXD1-KD+galectin-3-OE-induced M2 polarization, reducing CD206 and Arg-1 levels by approximately 70%, while restoring CD86 and iNOS levels to near NC group levels (Figure 7A). Western blot detection showed that the protein expression trends of p-STAT3 and PD-L1 were completely consistent with M2 markers: p-STAT3 and PD-L1 levels were significantly increased in the Notch1-OE group; FOXD1 knockdown reduced their expression; galectin-3 overexpression restored them; while addition of CD47 neutralizing antibody again reduced p-STAT3 and PD-L1 (Figure 7B). These results indicate that the CD47/SIRPα axis is required for Notch1-induced STAT3 activation and PD-L1 upregulation, and blocking this axis can reverse M2 polarization. The above results indicate that Notch1 induces TAM M2 polarization by activating the YAP/EZH2/JMJD3/PTEN/FOXD1/galectin-3/CD47 signaling pathway, thereby promoting NSCLC development.

### 3.8 Notch1 induces TAM M2 polarization through activation of the YAP/EZH2/JMJD3/PTEN/FOXD1/galectin-3/CD47 signaling pathway, affecting A549 cell apoptosis, proliferation, migration, and invasion, thereby promoting NSCLC development

**Figure 8:**
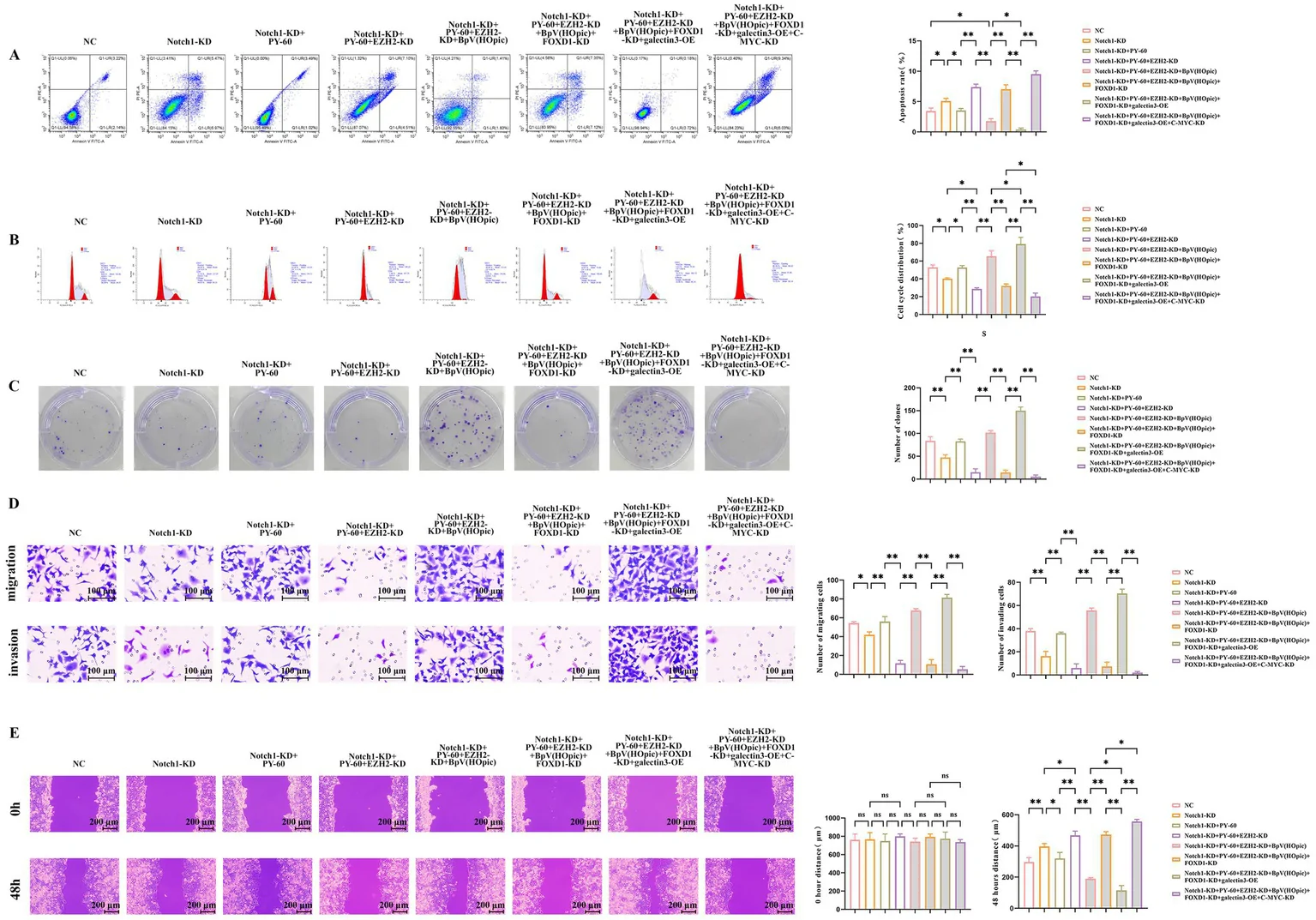
Notch1 induces TAM M2 polarization through activation of the YAP/EZH2/JMJD3/PTEN/FOXD1/galectin-3/CD47 signaling pathway, affecting A549 cell apoptosis, proliferation, migration, and invasion, thereby promoting NSCLC development. **(A)** Flow cytometry detection of A549 cell apoptosis after co-culture and statistical analysis of apoptosis rate. **(B)** Flow cytometry detection of A549 cell cycle after co-culture and statistical analysis of S-phase cell proportion. **(C)** Plate colony formation assay detection of A549 cell clonogenic ability after co-culture and statistical analysis of colony numbers. **(D)** Transwell assay detection of A549 cell invasion ability after co-culture and statistical analysis of invasive cell numbers. **(E)** Scratch assay detection of A549 cell migration ability after co-culture and statistical analysis of scratch width. Data are presented as mean ± standard deviation. *N* = 3, *p* < 0.05 indicates statistically significant differences, ns*P* > 0.05, *p < 0.05, **p < 0.01.

To evaluate the functional impact of TAM phenotypic changes on tumor cells, A549 cells from different treatment groups were co-cultured with THP-1 cells. Results showed that under conditions of Notch1 activation or PTEN inhibition, TAMs significantly inhibited A549 cell apoptosis (Figure 8A), promoted cell cycle progression (Figure 8B), colony formation (Figure 8C), migration (Figure 8D), and invasion (Figure 8E). Conversely, in Notch1 or EZH2 knockdown groups, TAMs exhibited inhibitory effects on tumor cell malignant phenotypes. This series of functional experiments confirms that Notch1-induced TAM M2 polarization is a key driver of NSCLC cell malignant behavior.

**Figure 9:**
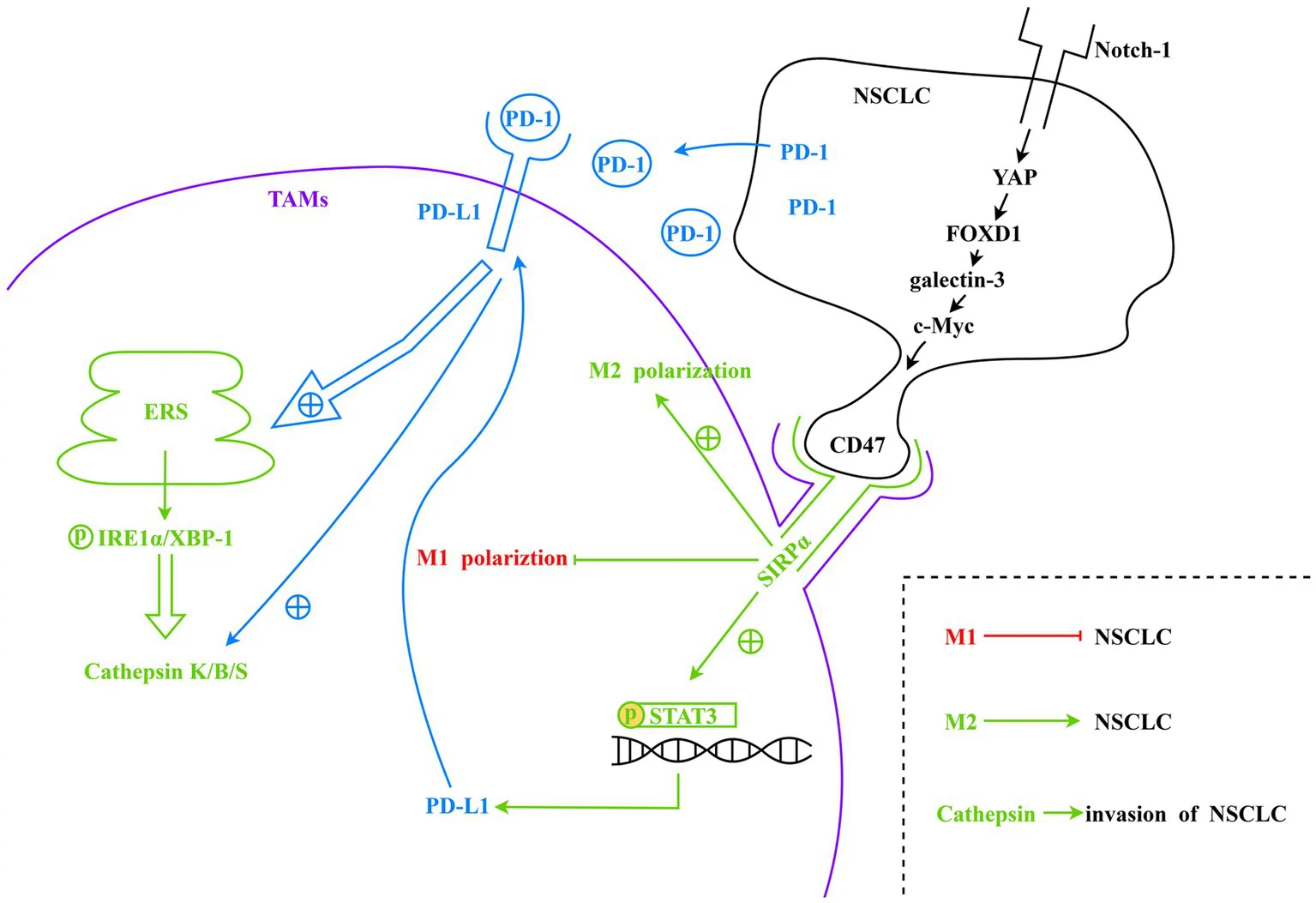
Schematic diagram of Notch1 inducing TAM M2 polarization through activation of the YAP/EZH2/JMJD3/PTEN/FOXD1/galectin-3/CD47 signaling pathway, thereby promoting NSCLC.

In summary, in NSCLC, tumor cells activate Notch1 signaling through Jagged-1, triggering NICD/RBP-J-mediated YAP synthesis and activation. Activated YAP enhances Jagged-1 expression through positive feedback, forming a Notch1/YAP signal amplification loop, and inhibits PTEN expression by activating EZH2 (in antagonism with JMJD3). PTEN deficiency relieves dual inhibition of the PI3K/Hippo pathways, leading to sustained YAP activation, which subsequently drives FOXD1 transcription and downstream expression of galectin-3 and C-MYC. C-MYC not only promotes tumor proliferation and invasion but also induces high expression of the immune checkpoint molecule CD47. CD47 binds to SIRPα on the surface of tumor-associated TAMs, blocking M1 polarization, driving M2 polarization, and activating the STAT3/PD-L1 axis. PD-L1 further induces ERS and promotes Cathepsin K/B expression. Notch1 induces TAM M2 polarization by regulating the YAP/EZH2/JMJD3/PTEN/FOXD1/galectin-3/CD47 signaling pathway, thereby promoting NSCLC progression (Figure 9). The original membranes and raw data of this study were provided in Supplementary Files 1, 2, respectively.

## 4 Discussion

The malignant progression of non-small cell lung cancer depends not only on the proliferation and invasion capabilities of tumor cells themselves, but also on the functional remodeling of immune cells within the tumor microenvironment, among which M2 polarization of tumor-associated macrophages is a core component of immune evasion. Through bioinformatics analysis, *in vivo* and *in vitro* functional experiments, and molecular mechanism validation, this study systematically reveals for the first time the complete molecular pathway by which Notch1 drives TAM M2 polarization through the YAP/EZH2/JMJD3/PTEN/FOXD1/galectin-3/CD47 signaling axis, and confirms the critical role of this axis in promoting NSCLC progression.

One of the core findings of this study is the identification of a positive feedback amplification loop between Notch1 and YAP. We demonstrated that in NSCLC cells, Notch1 activates YAP transcription through the classical NICD/RBP-J pathway, while activated YAP in turn enhances Jagged-1 expression, thereby continuously maintaining Notch1 signaling activity. This bidirectional regulatory mechanism enables tumor cells to rapidly amplify signaling cascades under TME stimuli such as hypoxia, conferring enhanced plasticity and adaptive capacity. This finding not only corroborates previously reported cross-talk between Notch and Hippo pathways (20), but further clarifies the functional output of this loop in immune microenvironment regulation.

At the epigenetic level, we found that YAP upregulates EZH2 and antagonizes JMJD3 function, leading to increased histone H3K27me3 modification and transcriptional repression of the tumor suppressor gene PTEN. The role of the EZH2/JMJD3 “writer-eraser” antagonistic pair in the Notch1-YAP pathway has not been fully described previously, and this study fills this gap. Previous studies have confirmed that YAP and TAZ can form nuclear complexes with EZH2 and MYC to transcriptionally repress tumor suppressors including PTEN (21). More importantly, we demonstrated that PTEN deficiency in NSCLC simultaneously relieves dual inhibition of both the PI3K/AKT pathway and the Hippo pathway: PTEN deficiency both activates AKT leading to LATS kinase inactivation and directly attenuates Hippo pathway kinase activity, resulting in sustained YAP nuclear translocation, forming a “PTEN deficiency/YAP activation/further PTEN suppression” vicious cycle. PTEN, as a key negative regulator of the PI3K/AKT pathway, whose deficiency or inactivation is closely associated with aberrant activation of this pathway in various cancers. This dual regulatory mechanism explains why YAP can exhibit highly persistent activation in NSCLC and provides a theoretical basis for combined targeting of PI3K and Hippo pathways.

Downstream of the signaling cascade, we demonstrated that YAP drives FOXD1 transcription by directly binding to its promoter, subsequently upregulating galectin-3 and C-MYC. ChIP-qPCR experiments provided direct evidence for this transcriptional regulatory relationship. Notably, galectin-3 serves as a critical bridge linking tumor cell-intrinsic programs and immune microenvironment signals in this pathway. On one hand, galectin-3 promotes tumor cell proliferation and invasion by enhancing C-MYC expression; on the other hand, C-MYC directly binds to the CD47 promoter to upregulate its expression, converting tumor-intrinsic oncogenic signals into immunosuppressive signals. The short title of this study highlights “Galectin-3 Induces Macrophage Immunosuppression by Regulating CD47/SIRP*α* Signaling,” precisely because galectin-3 occupies a hub position in this signaling axis, tightly coupling Notch1/YAP-driven transcriptional reprogramming with CD47-mediated macrophage functional remodeling.

CD47, as a “do not eat me” signal, upon binding to SIRPα on the macrophage surface, not only inhibits phagocytic function but is also demonstrated in this study to directly drive TAM polarization toward the M2 phenotype (22). Using a direct contact co-culture system to simulate membrane receptor-ligand interactions, we found that Notch1 signaling axis-activated CD47 through SIRPα significantly induced macrophage STAT3 phosphorylation and PD-L1 upregulation, while promoting M2 markers (CD206, Arg-1) and lysosomal protease Cathepsin K expression, and inhibiting M1 markers (CD86, iNOS) and TNF-α. Blockade of the CD47/SIRPα axis using SIRPα-Fc fusion protein almost completely reversed this polarization effect, indicating that CD47/SIRPα is the key execution node for Notch1 signaling-driven M2 polarization. Furthermore, PD-L1 upregulation further reinforces the immunosuppressive microenvironment and synergizes with STAT3 to induce endoplasmic reticulum stress and Cathepsin K/B expression, which may further promote tumor extracellular matrix remodeling and invasion. Flow cytometry analysis of subcutaneous xenograft tumor models further confirmed that Notch1 overexpression did not alter total macrophage infiltration numbers in tumor tissues, but significantly increased the M2/M1 ratio, suggesting that Notch1 primarily regulates functional polarization rather than chemotactic recruitment of macrophages.

Through virtual knockout analysis and stepwise pathway rescue experiments, this study validated the hierarchical relationships of this signaling axis at multiple levels. From Notch1 to YAP nuclear translocation, to epigenetic regulation of PTEN by EZH2/JMJD3, to stepwise activation of FOXD1/galectin-3/C-MYC and CD47, the experimental design employing a “knockdown-rescue-reknockdown” strategy clearly delineated the upstream-downstream relationships and causal connections at each node. This systematic pathway dissection provides a complete molecular framework for understanding the immunoregulatory functions of Notch1 in NSCLC.

At the clinical translational level, this study provides multiple potential targets for combination immunotherapy in NSCLC. First, targeting Notch1 or *γ*-secretase can simultaneously inhibit tumor cell-intrinsic proliferation and TAM M2 polarization, offering “two birds with one stone” potential. Second, CD47/SIRPα blocking antibodies have already demonstrated antitumor immune activity in multiple clinical trials, and this study reveals their potential synergy with anti-PD-L1 therapy by blocking the “CD47/STAT3/PD-L1” positive feedback loop to reshape TAM polarization status (23). Additionally, EZH2 inhibitors, YAP-TEAD inhibitors, or galectin-3 inhibitors can all serve as intervention nodes upstream of this signaling axis. Given the presence of multiple feedback loops in the pathway, combination blockade may be more effective than monotherapy in overcoming drug resistance.

However, this study has certain limitations. First, *in vitro* experiments were primarily based on A549 and THP-1 monocytic cell lines. Although THP-1-derived macrophages represent a classical model for studying TAM polarization, they cannot fully recapitulate the heterogeneity of primary monocytes/macrophages and TME complexity *in vivo*. Subsequent studies need to validate findings in primary monocyte and patient-derived organoid-immune cell co-culture systems. Second, the in vivo experiments employed nude mouse models, whose T-cell immunodeficiency may underestimate the role of the PD-L1 pathway in adaptive immunity. Immunocompetent mouse models or humanized mouse models would be more suitable for assessing the overall impact of this signaling axis on T-cell antitumor immunity. Furthermore, although this study used 1% O₂ hypoxic conditions to simulate the TME, hypoxia itself also affects macrophage polarization. Although we attempted to exclude this confounding factor through intergroup relative comparisons and normoxic control experiments, the in vivo oxygen gradient changes still require more refined simulation. Finally, although bioinformatics analysis and in vitro experiments strongly support the role of the Notch1-YAP axis in TAM polarization, direct validation of pathway protein expression and TAM polarization marker colocalization in clinical samples is lacking. Future studies using multiplex immunohistochemistry or spatial transcriptomics to simultaneously detect the spatial distribution relationship between the Notch1/YAP/EZH2/PTEN/CD47 axis and M2 macrophage markers in NSCLC tissue sections will further strengthen the clinical relevance of this mechanism.

In summary, this study is the first to completely elucidate the molecular mechanism by which Notch1 induces TAM M2 polarization through the YAP/EZH2/JMJD3/PTEN/FOXD1/galectin-3/CD47 signaling axis, revealing a multi-layered synergistic network through which tumor cells achieve immune evasion via epigenetic regulation, Hippo pathway inactivation, and immune checkpoint upregulation. This finding not only deepens our understanding of NSCLC immune microenvironment regulation, but also provides important experimental evidence and theoretical support for developing combination immunotherapy strategies targeting the Notch1-YAP-CD47 axis. Future research will focus on validating the therapeutic target value of this pathway in models more closely resembling physiological conditions, and exploring combination applications with existing immune checkpoint inhibitors.

## Data Availability

The raw data supporting the conclusions of this article will be made available by the authors, without undue reservation.

## References

[1] Bajbouj K Al-Ali A Ramakrishnan RK Saber-Ayad M Hamid Q. Histone modification in NSCLC: molecular mechanisms and therapeutic targets. Int J Mol Sci. (2021) 22:11701. doi: 10.3390/ijms222111701, 34769131 PMC8584007

[2] Desai A Peters S. Immunotherapy-based combinations in metastatic NSCLC. Cancer Treat Rev. (2023) 116:102545. doi: 10.1016/j.ctrv.2023.102545, 37030062

[3] Cheng K Cai N Zhu J Yang X Liang H Zhang W. Tumor-associated macrophages in liver cancer: from mechanisms to therapy. Cancer Commun. (2022) 42:1112–40. doi: 10.1002/cac2.12345, 36069342 PMC9648394

[4] Boutilier AJ Elsawa SF. Macrophage polarization states in the tumor microenvironment. Int J Mol Sci. (2021) 22:6995. doi: 10.3390/ijms22136995, 34209703 PMC8268869

[5] Zanotti S Canalis E. Notch Signaling and the skeleton. Endocr Rev. (2016) 37:223–53. doi: 10.1210/er.2016-1002, 27074349 PMC4890266

[6] Yan W Menjivar RE Bonilla ME Steele NG Kemp SB du W . Notch Signaling regulates immunosuppressive tumor-associated macrophage function in pancreatic cancer. Cancer Immunol Res. (2024) 12:91–106. doi: 10.1158/2326-6066.CIR-23-0037, 37931247 PMC10842043

[7] Sharma R Sharma S Shriwas P Mehta L Vu AH Mouw JK . Intra-tumoral YAP and TAZ heterogeneity drives collective NSCLC invasion that is targeted by SUMOylation inhibitor TAK-981. iScience. (2024) 27:111133. doi: 10.1016/j.isci.2024.111133, 39524367 PMC11544388

[8] Yang Y Ni M Zong R Yu M Sun Y Li J . Targeting Notch1-YAP circuit reprograms macrophage polarization and alleviates acute liver injury in mice. Cell Mol Gastroenterol Hepatol. (2023) 15:1085–104. doi: 10.1016/j.jcmgh.2023.01.002, 36706917 PMC10036742

[9] Zhou X Chen H Hu Y Ma X Li J Shi Y . Enhancer of zeste homolog 2 promotes renal fibrosis after acute kidney injury by inducing epithelial-mesenchymal transition and activation of M2 macrophage polarization. Cell Death Dis. (2023) 14:253. doi: 10.1038/s41419-023-05782-4, 37029114 PMC10081989

[10] Yu C Xiong C Tang J Hou X Liu N Bayliss G . Histone demethylase JMJD3 protects against renal fibrosis by suppressing TGFβ and notch signaling and preserving PTEN expression. Theranostics. (2021) 11:2706–21. doi: 10.7150/thno.48679, 33456568 PMC7806480

[11] Sadrkhanloo M Paskeh MDA Hashemi M Raesi R Bahonar A Nakhaee Z . New emerging targets in osteosarcoma therapy: PTEN and PI3K/Akt crosstalk in carcinogenesis. Pathol Res Pract. (2023) 251:154902. doi: 10.1016/j.prp.2023.154902, 37922723

[12] Orozco-García E Van Meurs DJ Calderón JC Narvaez-Sanchez R Harmsen MC. Endothelial plasticity across PTEN and hippo pathways: a complex hormetic rheostat modulated by extracellular vesicles. Transl Oncol. (2023) 31:101633. doi: 10.1016/j.tranon.2023.101633, 36905871 PMC10020115

[13] Yang X Wen J He Q Wang S Ruan Q Liao Q . MicroRNA3650 promotes gastric Cancer proliferation and migration through the PTEN/PI3K-AKT-mTOR and hippo pathways. Protein Pept Lett. (2023) 30:966–73. doi: 10.2174/0109298665265642231020043809, 38031771

[14] Jia X Yan B Tian X Liu Q Jin J Shi J . CD47/SIRPα pathway mediates cancer immune escape and immunotherapy. Int J Biol Sci. (2021) 17:3281–7. doi: 10.7150/ijbs.60782, 34512146 PMC8416724

[15] Fan Y Song S Li Y Dhar SS Jin J Yoshimura K . Galectin-3 cooperates with CD47 to suppress phagocytosis and T-cell immunity in gastric Cancer peritoneal metastases. Cancer Res. (2023) 83:3726–38. doi: 10.1158/0008-5472.CAN-23-0783, 37738407 PMC10843008

[16] Fu L Hu Y Song M Liu Z Zhang W Yu FX . Up-regulation of FOXD1 by YAP alleviates senescence and osteoarthritis. PLoS Biol. (2019) 17:e3000201. doi: 10.1371/journal.pbio.3000201, 30933975 PMC6459557

[17] Li CH Chang YC Hsiao M Liang SM. FOXD1 and Gal-3 form a positive regulatory loop to regulate lung cancer aggressiveness. Cancers. (2019) 11:1897. doi: 10.3390/cancers11121897, 31795213 PMC6966623

[18] Brustugun OT. Hypoxia as a cause of treatment failure in non–small cell carcinoma of the lung. Semin Radiat Oncol. (2015) 25:87–92. doi: 10.1016/j.semradonc.2014.11.006 25771412

[19] Levallet J Biojout T Bazille C Douyère M Dubois F Ferreira DL . Hypoxia-induced activation of NDR2 underlies brain metastases from non-small cell lung cancer. Cell Death Dis. (2023) 14:823. doi: 10.1038/s41419-023-06345-3 38092743 PMC10719310

[20] Moya IM Halder G. Hippo–YAP/TAZ signalling in organ regeneration and regenerative medicine. Nat Rev Mol Cell Biol. (2019) 20:211–26. doi: 10.1038/s41580-018-0086-y, 30546055

[21] Lo Sardo F Turco C Messina B Sacconi A Auciello FR Pulito C . The oncogenic axis YAP/MYC/EZH2 impairs PTEN tumor suppression activity enhancing lung tumorigenicity. Cell Death Discov. (2024) 10:452. doi: 10.1038/s41420-024-02216-8, 39455556 PMC11511861

[22] Shen Y Li C Zhou L Huang JA. G protein-coupled oestrogen receptor promotes cell growth of non-small cell lung cancer cells via YAP1/QKI/circNOTCH1/m6A methylated NOTCH1 signalling. J Cell Mol Med. (2021) 25:284–96. doi: 10.1111/jcmm.15997, 33237585 PMC7810948

[23] Charbat MA Abdulhalim YH Alrabeei MA Abdo Hassan W. Role of Notch1 signaling pathway in small cell lung carcinoma. Iran J Pathol. (2024) 19:365–75. doi: 10.30699/IJP.2024.2013339.3184 40034926 PMC11872034

