# Supplementary material for: Notch-1 induces M2 polarization of tumor-associated macrophages via the YAP/EZH2/FOXD1/CD47 signaling axis, thereby driving non-small cell lung cancer progression
Source: Front Med (Lausanne). 2026 Aug 10;13:1801942. doi: 10.3389/fmed.2026.1801942 (PMC13500596; doi:10.3389/fmed.2026.1801942)

Supplementary file 1: Original strip chart of western blot for this study

Fig5A

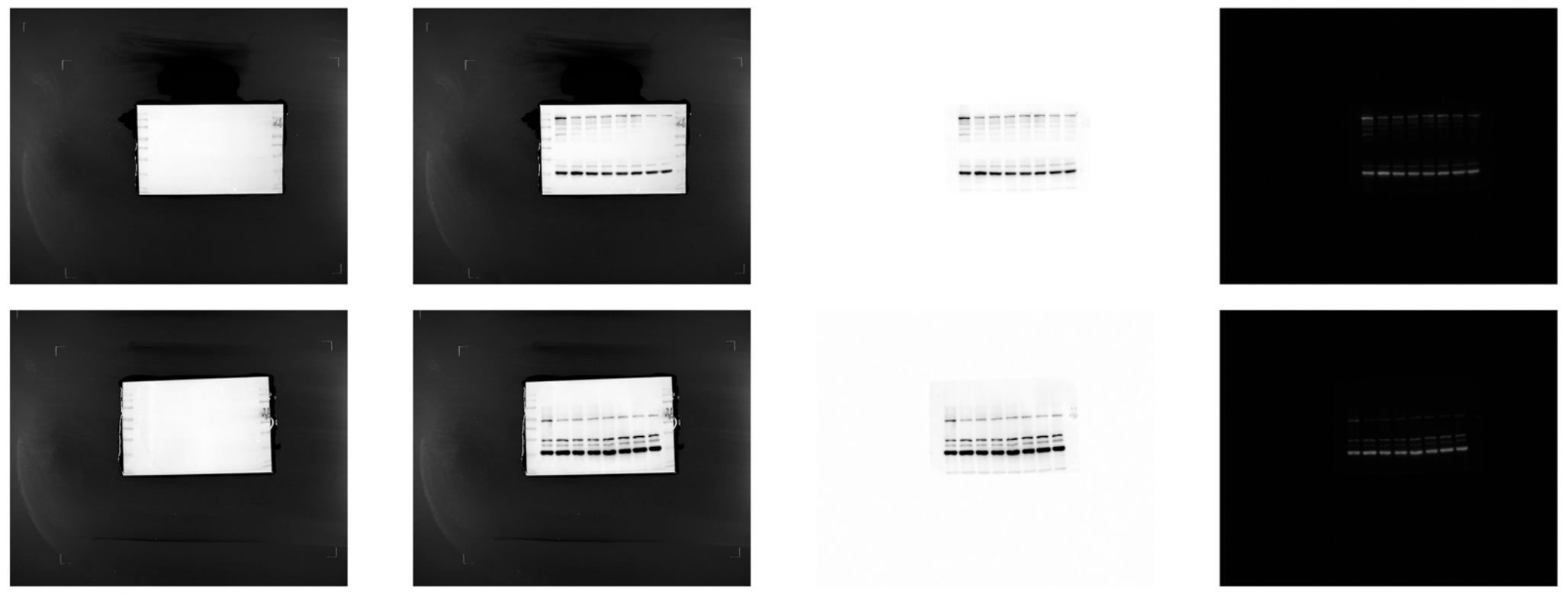

Fig5B

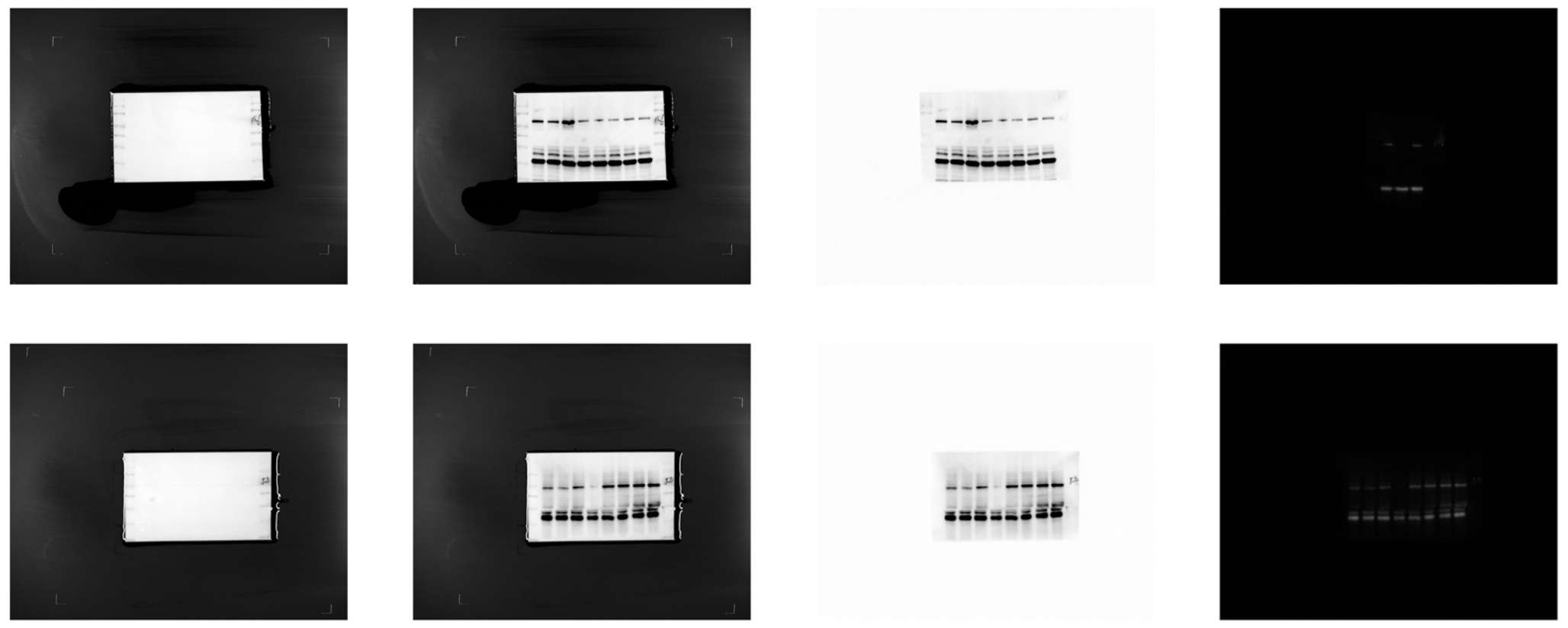

Fig6B

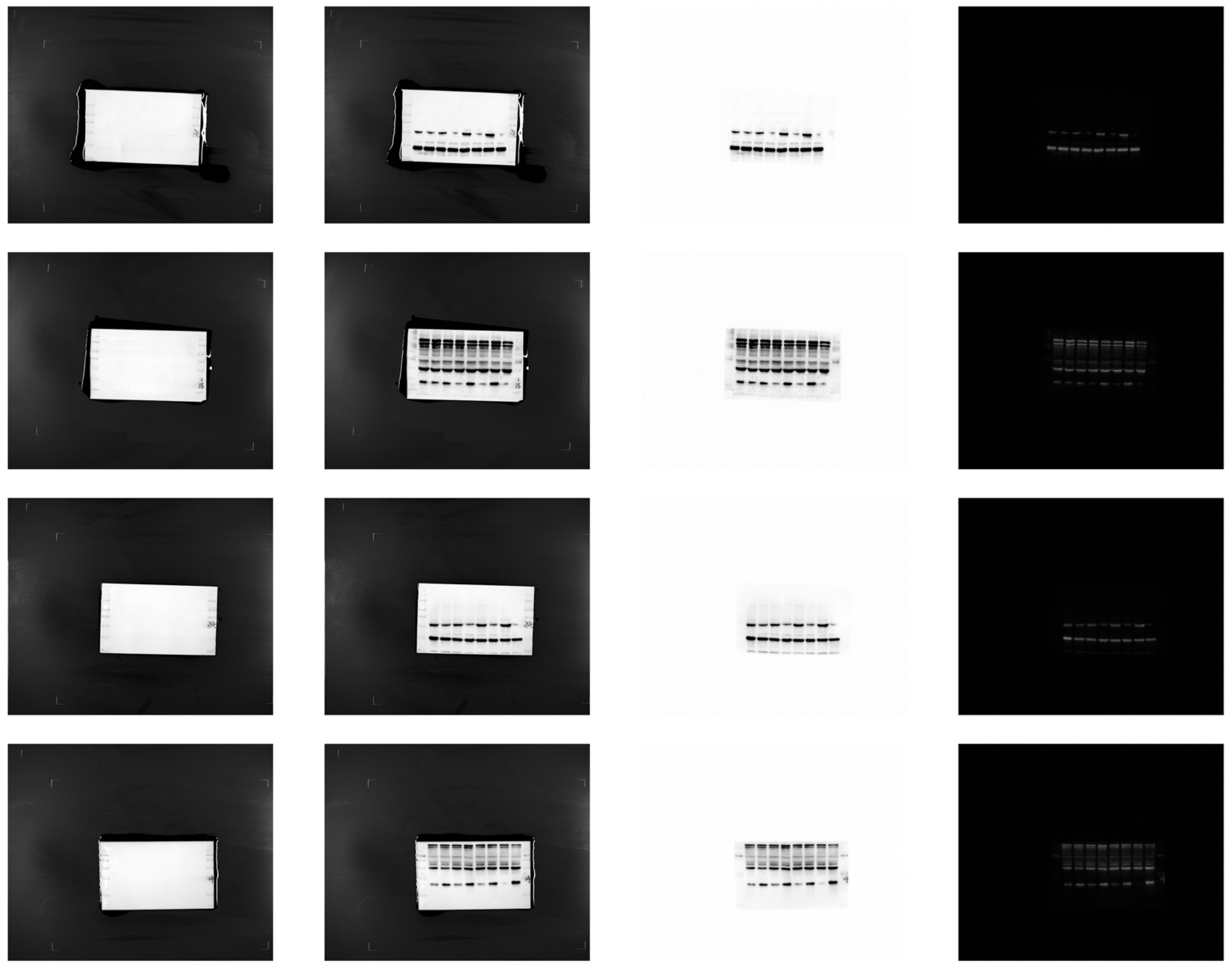

Fig6A

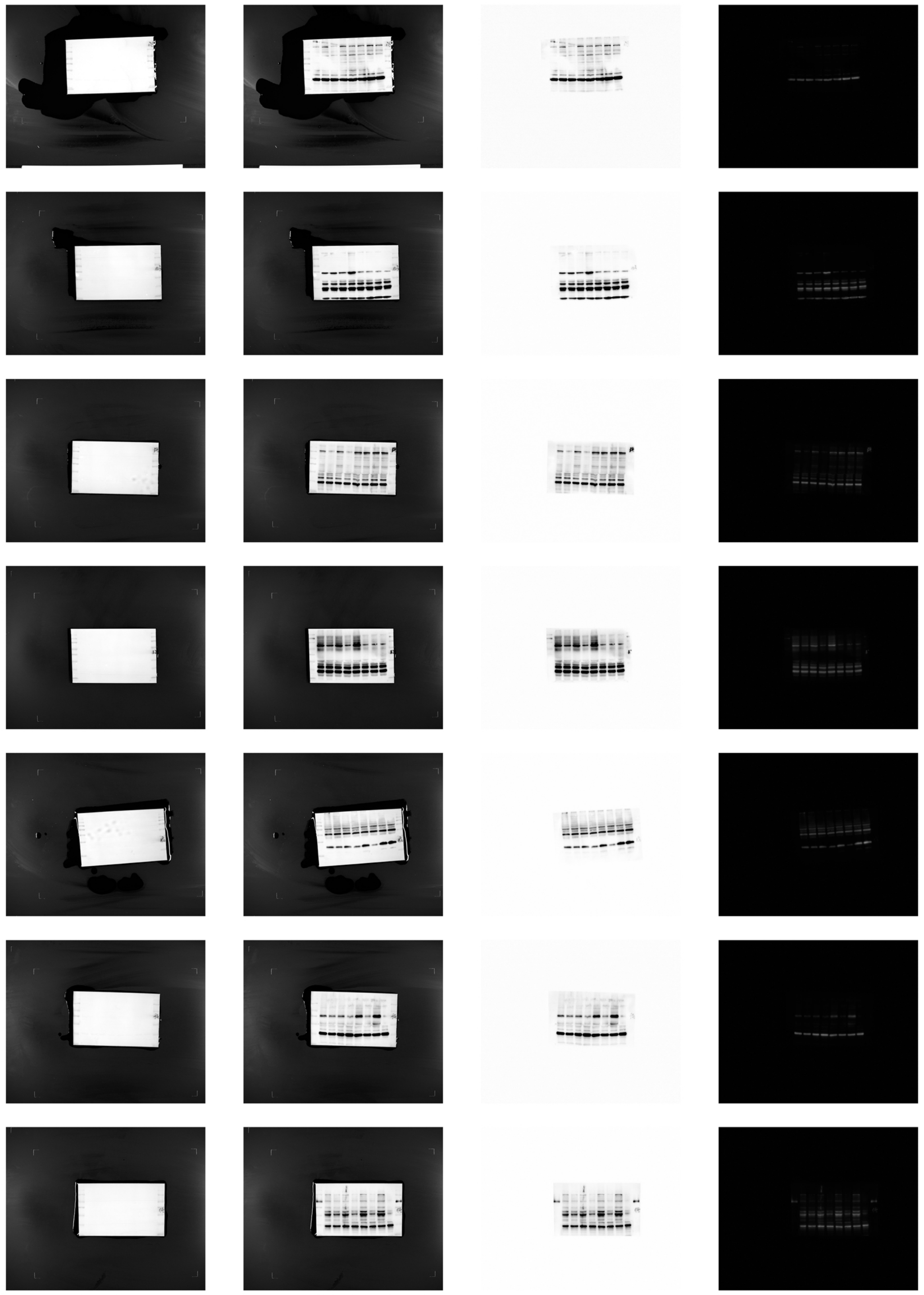

Fig7B

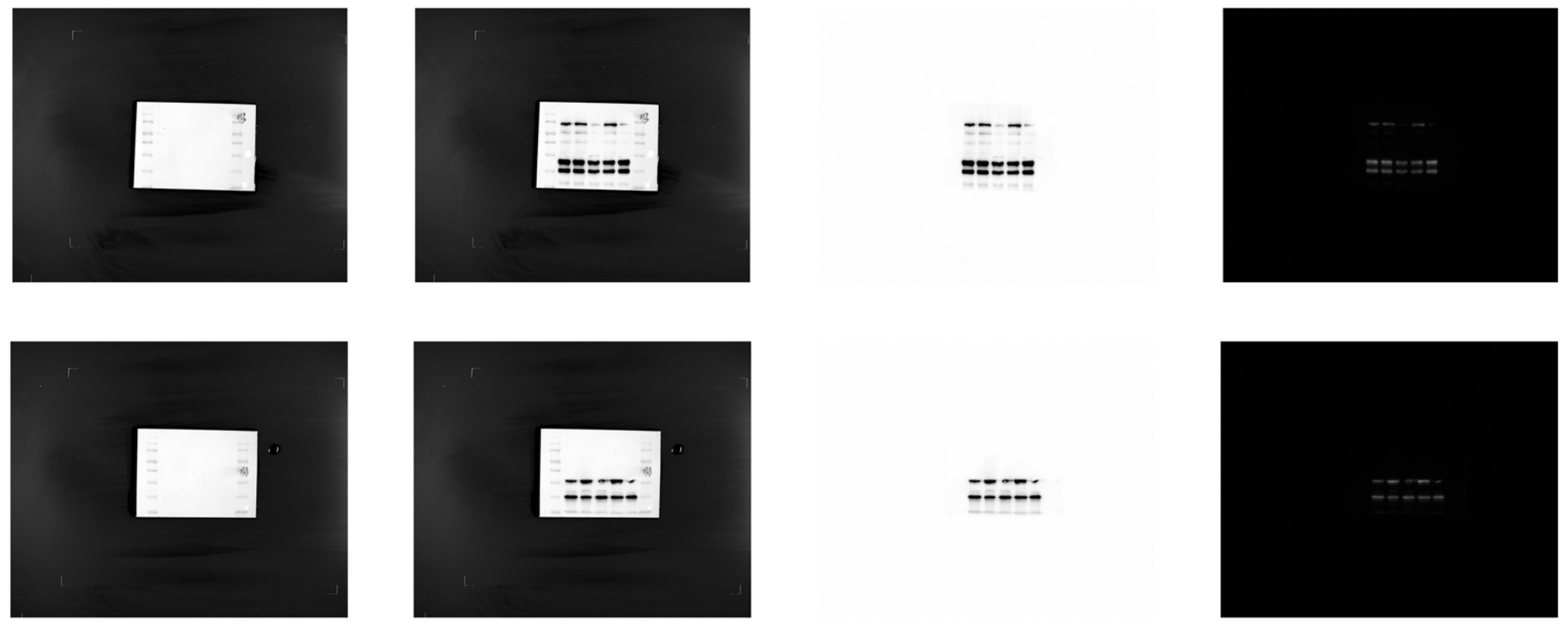

Supplement: Supplementary file 1 [file Data_Sheet_1.PDF]
