# Supplementary material for: Notch-1 induces M2 polarization of tumor-associated macrophages via the YAP/EZH2/FOXD1/CD47 signaling axis, thereby driving non-small cell lung cancer progression
Source: Front Med (Lausanne). 2026 Aug 10;13:1801942. doi: 10.3389/fmed.2026.1801942 (PMC13500596; doi:10.3389/fmed.2026.1801942)
Supplement: Supplementary file 2 [file Table_1.docx]

Supplementary Table S1: Key Antibodies, Primers, Lentiviral Constructs, and Inhibitor Information

| Category | Name | Catalog/Source | Dilution/Concentration | Purpose |
| --- | --- | --- | --- | --- |
| **Antibodies (WB)** | Notch1 | Abcam ab52627 | 1:1000 | Detect total Notch1 protein |
|  | YAP | Abcam ab52771 | 1:2000 | Detect YAP |
|  | EZH2 | Abcam ab287893 | 1:1000 | Detect EZH2 |
|  | JMJD3 | Abcam ab38113 | 1:1000 | Detect JMJD3 |
|  | PTEN | Abcam ab267787 | 1:2000 | Detect PTEN |
|  | p‑PI3K (Tyr458) | Abcam ab138364 | 1:500 | Detect p‑PI3K |
|  | FOXD1 | ThermoFisher PA5‑35145 | 1:500 | Detect FOXD1 |
|  | Galectin‑3 | Abcam ab76245 | 1:2000 | Detect Galectin‑3 |
|  | C‑MYC | Abcam ab32072 | 1:1000 | Detect c‑Myc |
|  | CD47 | Abcam ab218810 | 1:1000 | Detect CD47 |
|  | Arginase‑1 | Abcam ab133543 | 1:1000 | M2 marker |
|  | TNF‑α | Abcam ab183218 | 1:1000 | M1 marker |
|  | PD‑L1 | Abcam ab213524 | 1:1000 | Immune checkpoint |
|  | Cathepsin K | Abcam ab187647 | 1:1000 | Lysosomal protease |
|  | GAPDH | Abcam ab8245 | 1:5000 | Loading control |
| **Antibodies (IHC/IF)** | F4/80 | Abcam ab6640 | 1:200 | Macrophage |
|  | CD68 | Abcam ab125212 | 1:200 | Macrophage |
|  | CD86 | Abcam ab239075 | 1:100 | M1 marker |
|  | iNOS | Abcam ab178945 | 1:100 | M1 marker |
|  | CD163 | Abcam ab182422 | 1:200 | M2 marker |
|  | CD206 | Abcam ab64693 | 1:200 | M2 marker |
| **Secondary antibodies** | Anti‑rabbit HRP | Abcam ab6721 | 1:5000 | WB |
|  | Anti‑mouse HRP | Abcam ab6789 | 1:5000 | WB |
|  | Alexa Fluor 488/594 | ThermoFisher | 1:500 | IF |
| **Primers (qRT‑PCR)** | IL‑1β‑F | 5'‑ATGCCTCGTGCTGTCTGACC‑3' | 10 μM |  |
|  | IL‑1β‑R | 5'‑TTTGTCGTTGCTTGTCTCTCCTTG‑3' | 10 μM |  |
|  | TNF‑α‑F | 5'‑CACCACGCTCTTCTGTCTACTGAAC‑3' | 10 μM |  |
|  | TNF‑α‑R | 5'‑TGACGGCAGAGAGGAGGTTGAC‑3' | 10 μM |  |
|  | Arginase‑1‑F | 5'‑TTGGGGTGGGATGCTCACACTG‑3' | 10 μM |  |
|  | Arginase‑1‑R | 5'‑GTACACGATGTCTTTGGCAGA‑3' | 10 μM |  |
|  | Cathepsin B‑F | 5'‑GCAGGCCGGGCACAAC‑3' | 10 μM |  |
|  | Cathepsin B‑R | 5'‑GGAGGCCCAGAGAGCTGCCACAT‑3' | 10 μM |  |
|  | Cathepsin K‑F | 5'‑ATGTGAACCATGCAGTGTTGGTGG‑3' | 10 μM |  |
|  | Cathepsin K‑R | 5'‑ATGCCGCAGGCGTTGTTCTTATTC‑3' | 10 μM |  |
|  | β‑actin‑F | 5'‑CTATCGGCAATGAGCGGTTCC‑3' | 10 μM |  |
|  | β‑actin‑R | 5'‑GCACTGTGTTGGCATAGAGGTC‑3' | 10 μM |  |
| **Lentiviral constructs** | Notch1‑OE | pLVX‑Puro‑Notch1 (Hanheng Bio) | – | Overexpression |
|  | galectin‑3‑OE | pLVX‑Puro‑galectin‑3 (Hanheng Bio) | – | Overexpression |
|  | Notch1‑KD | pLKO.1‑shNotch1 (Hanheng Bio) | – | Knockdown |
|  | EZH2‑KD | pLKO.1‑shEZH2 (Hanheng Bio) | – | Knockdown |
|  | FOXD1‑KD | pLKO.1‑shFOXD1 (Hanheng Bio) | – | Knockdown |
|  | C‑MYC‑KD | pLKO.1‑shC‑MYC (Hanheng Bio) | – | Knockdown |
|  | CD47‑KD | pLKO.1‑shCD47 (Hanheng Bio) | – | Knockdown |
| **Inhibitors/Agonists** | PY‑60 (YAP agonist) | MedChemExpress HY‑123456 | 0.7 μM | YAP activation |
|  | BpV(HOpic) (PTEN inhibitor) | MedChemExpress HY‑123457 | 8 nM | PTEN inhibition |
|  | PMA | Sigma P8139 | 100 ng/mL | THP‑1 differentiation |
|  | CD47 neutralizing antibody | BioXCell BE0283 (clone B6H12) | 10 μg/mL | Blockade assay |
|  | SIRPα‑Fc | R&D Systems 4546‑TA | 10 μg/mL | Blockade assay |
| **Kits** | RIPA lysis buffer | Merck 20‑188 | – | Protein extraction |
|  | BCA kit | Sigma BCA1 | – | Protein quantification |
|  | Trizol | Invitrogen 15596026 | – | RNA extraction |
|  | PrimeScript RT kit | Takara 047A | – | Reverse transcription |
|  | TB Green qPCR mix | Takara 820A | – | qPCR |
| **ChIP antibodies** | Anti‑YAP | Cell Signaling 8579 | – | ChIP‑qPCR |
|  | Anti‑C‑MYC | Abcam ab32072 | – | ChIP‑qPCR |
|  | Normal Rabbit IgG | Cell Signaling 2729 | – | ChIP‑qPCR negative control |
| **ChIP primers** | FOXD1 Pro‑F | 5'‑GCCGCTGTGTGTTCTGTGAG‑3' | – | ChIP‑qPCR |
|  | FOXD1 Pro‑R | 5'‑GCTGCCGCGAGTTCTGTCT‑3' | – | ChIP‑qPCR |
|  | CD47 Pro‑F | 5'‑CGCCGCCCCTCTCTACTTAA‑3' | – | ChIP‑qPCR |
|  | CD47 Pro‑R | 5'‑CGGGATTTAGGGTGGGTTCT‑3' | – | ChIP‑qPCR |
